# Supplementary material for: Population structure of three New Zealand crested penguins identifies current conservation challenges for the Fiordland penguin/tawaki, erect-crested penguin, and eastern rockhopper penguin
Source: PLoS One. 2025 Aug 27;20(8):e0329545. doi: 10.1371/journal.pone.0329545 (PMC12385409; doi:10.1371/journal.pone.0329545)
Supplement: S1 Table — Full list of tawaki (Eudyptes pachyrhynchus), erect-crested penguin (Eudyptes sclateri), and eastern rockhopper penguin (Eudyptes filholi) samples included in the analysis. (DOCX) [file pone.0329545.s001.docx]

**S1 Table. List of all samples and sampling locations included**. Full list of tawaki (*Eudyptes pachyrhynchus*), erect-crested penguin (*Eudyptes sclateri*), and eastern rockhopper penguin (*Eudyptes filholi*) samples included in the analysis.

| Sample ID | Species | Collection Year | Site | Colony | Latitude | Longitude | Method |
| --- | --- | --- | --- | --- | --- | --- | --- |
| AB-R 02 | *E. filholi* | 2022 | Antipodes Island | Anchorage Bay | –49.6680 | 178.8079 | DNeasy |
| AB-R 04 | *E. filholi* | 2022 | Antipodes Island | Anchorage Bay | –49.6680 | 178.8079 | DNeasy |
| AB-R 06 | *E. filholi* | 2022 | Antipodes Island | Anchorage Bay | –49.6680 | 178.8079 | DNeasy |
| AB-R 07 | *E. filholi* | 2022 | Antipodes Island | Anchorage Bay | –49.6680 | 178.8079 | DNeasy |
| AB-R 09 | *E. filholi* | 2022 | Antipodes Island | Anchorage Bay | –49.6680 | 178.8079 | DNeasy |
| AB-R 11 | *E. filholi* | 2022 | Antipodes Island | Anchorage Bay | –49.6680 | 178.8079 | DNeasy |
| AB-R 13 | *E. filholi* | 2022 | Antipodes Island | Anchorage Bay | –49.6680 | 178.8079 | DNeasy |
| AB-R 14 | *E. filholi* | 2022 | Antipodes Island | Anchorage Bay | –49.6680 | 178.8079 | DNeasy |
| SC-R 02 | *E. filholi* | 2022 | Antipodes Island | South Coast | –49.7063 | 178.7483 | DNeasy |
| SC-R 04 | *E. filholi* | 2022 | Antipodes Island | South Coast | –49.7063 | 178.7483 | DNeasy |
| SC-R 05 | *E. filholi* | 2022 | Antipodes Island | South Coast | –49.7063 | 178.7483 | DNeasy |
| SC-R 07 | *E. filholi* | 2022 | Antipodes Island | South Coast | –49.7063 | 178.7483 | DNeasy |
| SC-R 08 | *E. filholi* | 2022 | Antipodes Island | South Coast | –49.7063 | 178.7483 | DNeasy |
| SC-R 09 | *E. filholi* | 2022 | Antipodes Island | South Coast | –49.7063 | 178.7483 | DNeasy |
| SC-R 11 | *E. filholi* | 2022 | Antipodes Island | South Coast | –49.7063 | 178.7483 | DNeasy |
| SC-R 14 | *E. filholi* | 2022 | Antipodes Island | South Coast | –49.7063 | 178.7483 | DNeasy |
| SC-R 15 | *E. filholi* | 2022 | Antipodes Island | South Coast | –49.7063 | 178.7483 | DNeasy |
| p14542 | *E. pachyrhynchus* | 2022 | Milford Sound | Harrison Cove | –44.6229 | 167.9119 | DNeasy |
| p14828 | *E. pachyrhynchus* | 2022 | Milford Sound | Harrison Cove | –44.6229 | 167.9119 | DNeasy |
| p14844 | *E. pachyrhynchus* | 2022 | Milford Sound | Harrison Cove | –44.6229 | 167.9119 | DNeasy |
| p15051 | *E. pachyrhynchus* | 2022 | Doubtful Sound | East Shelter Island | –45.2757 | 166.8975 | DNeasy |
| p15241 | *E. pachyrhynchus* | 2022 | Doubtful Sound | East Shelter Island | –45.2757 | 166.8975 | DNeasy |
| p15828 | *E. pachyrhynchus* | 2022 | Milford Sound | Harrison Cove | –44.6229 | 167.9119 | DNeasy |
| p17217 | *E. pachyrhynchus* | 2022 | Doubtful Sound | East Shelter Island | –45.2757 | 166.8975 | DNeasy |
| p17288 | *E. pachyrhynchus* | 2022 | Doubtful Sound | Seymour Island | –45.3079 | 167.0068 | DNeasy |
| p20806 | *E. pachyrhynchus* | 2022 | Doubtful Sound | East Shelter Island | –45.2757 | 166.8975 | DNeasy |
| p23986 | *E. pachyrhynchus* | 2022 | Doubtful Sound | Seymour Island | –45.3079 | 167.0068 | DNeasy |
| p24235 | *E. pachyrhynchus* | 2022 | Doubtful Sound | East Shelter Island | –45.2757 | 166.8975 | DNeasy |
| p32718 | *E. pachyrhynchus* | 2022 | Milford Sound | Harrison Cove | –44.6229 | 167.9119 | DNeasy |
| p38240 | *E. pachyrhynchus* | 2022 | Doubtful Sound | Seymour Island | –45.3079 | 167.0068 | DNeasy |
| p38700 | *E. pachyrhynchus* | 2022 | Doubtful Sound | East Shelter Island | –45.2757 | 166.8975 | DNeasy |
| p39923 | *E. pachyrhynchus* | 2022 | Doubtful Sound | Seymour Island | –45.3079 | 167.0068 | DNeasy |
| p40192 | *E. pachyrhynchus* | 2022 | Doubtful Sound | East Shelter Island | –45.2757 | 166.8975 | DNeasy |
| p40301 | *E. pachyrhynchus* | 2022 | Doubtful Sound | Seymour Island | –45.3079 | 167.0068 | DNeasy |
| p40348 | *E. pachyrhynchus* | 2022 | Doubtful Sound | Seymour Island | –45.3079 | 167.0068 | DNeasy |
| p40650 | *E. pachyrhynchus* | 2022 | Doubtful Sound | East Shelter Island | –45.2757 | 166.8975 | DNeasy |
| p40862 | *E. pachyrhynchus* | 2022 | Milford Sound | Harrison Cove | –44.6229 | 167.9119 | DNeasy |
| p46241 | *E. pachyrhynchus* | 2022 | Milford Sound | Harrison Cove | –44.6229 | 167.9119 | DNeasy |
| p46440 | *E. pachyrhynchus* | 2022 | Doubtful Sound | East Shelter Island | –45.2757 | 166.8975 | DNeasy |
| p46578 | *E. pachyrhynchus* | 2022 | Doubtful Sound | East Shelter Island | –45.2757 | 166.8975 | DNeasy |
| p46638 | *E. pachyrhynchus* | 2022 | Doubtful Sound | East Shelter Island | –45.2757 | 166.8975 | DNeasy |
| p46832 | *E. pachyrhynchus* | 2022 | Milford Sound | Harrison Cove | –44.6229 | 167.9119 | DNeasy |
| p54010 | *E. pachyrhynchus* | 2022 | Doubtful Sound | Seymour Island | –45.3079 | 167.0068 | DNeasy |
| p61917 | *E. pachyrhynchus* | 2022 | Doubtful Sound | East Shelter Island | –45.2757 | 166.8975 | DNeasy |
| p94352 | *E. pachyrhynchus* | 2022 | Doubtful Sound | East Shelter Island | –45.2757 | 166.8975 | DNeasy |
| EP 128 | *E. pachyrhynchus* | 2018 | West Coast | Jackson Head | –43.9629 | 168.6115 | Phenol |
| EP 129 | *E. pachyrhynchus* | 2018 | West Coast | Jackson Head | –43.9629 | 168.6115 | Phenol |
| EP 134 | *E. pachyrhynchus* | 2018 | West Coast | Jackson Head | –43.9629 | 168.6115 | Phenol |
| EP 137 | *E. pachyrhynchus* | 2018 | West Coast | Jackson Head | -43.9629 | 168.6115 | Phenol |
| EP 138 | *E. pachyrhynchus* | 2018 | West Coast | Jackson Head | –43.9629 | 168.6115 | Phenol |
| EP 140 | *E. pachyrhynchus* | 2018 | West Coast | Jackson Head | –43.9629 | 168.6115 | Phenol |
| EP 141 | *E. pachyrhynchus* | 2018 | West Coast | Jackson Head | –43.9629 | 168.6115 | Phenol |
| EP 142 | *E. pachyrhynchus* | 2018 | West Coast | Jackson Head | –43.9629 | 168.6115 | Phenol |
| EP 144 | *E. pachyrhynchus* | 2018 | Milford Sound | Harrison Cove | –44.6229 | 167.9119 | Phenol |
| EP 148 | *E. pachyrhynchus* | 2018 | Milford Sound | Harrison Cove | –44.6229 | 167.9119 | Phenol |
| EP 149 | *E. pachyrhynchus* | 2018 | Milford Sound | Harrison Cove | –44.6229 | 167.9119 | Phenol |
| EP 152 | *E. pachyrhynchus* | 2018 | Milford Sound | Harrison Cove | –44.6229 | 167.9119 | Phenol |
| EP 153 | *E. pachyrhynchus* | 2018 | Milford Sound | Harrison Cove | –44.6229 | 167.9119 | Phenol |
| EP 154 | *E. pachyrhynchus* | 2018 | Milford Sound | Harrison Cove | –44.6229 | 167.9119 | Phenol |
| EP 156 | *E. pachyrhynchus* | 2018 | Foveaux Strait | Whenua Hou | –46.7603 | 167.6408 | Phenol |
| EP 158 | *E. pachyrhynchus* | 2018 | Foveaux Strait | Whenua Hou | –46.7603 | 167.6408 | Phenol |
| EP 159 | *E. pachyrhynchus* | 2018 | Foveaux Strait | Whenua Hou | –46.7603 | 167.6408 | Phenol |
| EP 161 | *E. pachyrhynchus* | 2018 | Foveaux Strait | Whenua Hou | –46.7603 | 167.6408 | Phenol |
| EP 166 | *E. pachyrhynchus* | 2018 | Foveaux Strait | Whenua Hou | –46.7603 | 167.6408 | Phenol |
| EP 167 | *E. pachyrhynchus* | 2018 | Foveaux Strait | Whenua Hou | –46.7603 | 167.6408 | Phenol |
| EP 185 | *E. pachyrhynchus* | 2018 | Foveaux Strait | Whenua Hou | –46.7603 | 167.6408 | Phenol |
| HC 019 | *E. pachyrhynchus* | 2017 | Milford Sound | Harrison Cove | –44.6229 | 167.9119 | Phenol |
| JH 022 | *E. pachyrhynchus* | 2017 | West Coast | Jackson Head | –43.9629 | 168.6115 | Phenol |
| JH 024 | *E. pachyrhynchus* | 2017 | West Coast | Jackson Head | –43.9629 | 168.6115 | Phenol |
| JH 025 | *E. pachyrhynchus* | 2017 | West Coast | Jackson Head | –43.9629 | 168.6115 | Phenol |
| JH 028 | *E. pachyrhynchus* | 2017 | West Coast | Jackson Head | –43.9629 | 168.6115 | Phenol |
| JH 035 | *E. pachyrhynchus* | 2018 | West Coast | Jackson Head | –43.9629 | 168.6115 | Phenol |
| AB-E 01 | *E. sclateri* | 2022 | Antipodes Island | Anchorage Bay | –49.6680 | 178.8079 | DNeasy |
| AB-E 02 | *E. sclateri* | 2022 | Antipodes Island | Anchorage Bay | –49.6680 | 178.8079 | DNeasy |
| AB-E 03 | *E. sclateri* | 2022 | Antipodes Island | Anchorage Bay | –49.6680 | 178.8079 | DNeasy |
| AB-E 04 | *E. sclateri* | 2022 | Antipodes Island | Anchorage Bay | –49.6680 | 178.8079 | DNeasy |
| AB-E 05 | *E. sclateri* | 2022 | Antipodes Island | Anchorage Bay | –49.6680 | 178.8079 | DNeasy |
| AB-E 06 | *E. sclateri* | 2022 | Antipodes Island | Anchorage Bay | –49.6680 | 178.8079 | DNeasy |
| AB-E 07 | *E. sclateri* | 2022 | Antipodes Island | Anchorage Bay | –49.6680 | 178.8079 | DNeasy |
| AB-E 08 | *E. sclateri* | 2022 | Antipodes Island | Anchorage Bay | –49.6680 | 178.8079 | DNeasy |
| AB-E 09 | *E. sclateri* | 2022 | Antipodes Island | Anchorage Bay | –49.6680 | 178.8079 | DNeasy |
| AB-E 11 | *E. sclateri* | 2022 | Antipodes Island | Anchorage Bay | –49.6680 | 178.8079 | DNeasy |
| AB-E 12 | *E. sclateri* | 2022 | Antipodes Island | Anchorage Bay | –49.6680 | 178.8079 | DNeasy |
| AB-E 13 | *E. sclateri* | 2022 | Antipodes Island | Anchorage Bay | –49.6680 | 178.8079 | DNeasy |
| AB-E 14 | *E. sclateri* | 2023 | Antipodes Island | Anchorage Bay | –49.6680 | 178.8079 | DNeasy |
| AB-E 15 | *E. sclateri* | 2022 | Antipodes Island | Anchorage Bay | –49.6680 | 178.8079 | DNeasy |
| BI-E 01 | *E. sclateri* | 2022 | Bounty Islands | Proclamation Island | –47.7496 | 179.0259 | DNeasy |
| BI-E 02 | *E. sclateri* | 2022 | Bounty Islands | Proclamation Island | –47.7496 | 179.0259 | DNeasy |
| BI-E 03 | *E. sclateri* | 2022 | Bounty Islands | Proclamation Island | –47.7496 | 179.0259 | DNeasy |
| BI-E 04 | *E. sclateri* | 2022 | Bounty Islands | Proclamation Island | –47.7496 | 179.0259 | DNeasy |
| BI-E 05 | *E. sclateri* | 2022 | Bounty Islands | Proclamation Island | –47.7496 | 179.0259 | DNeasy |
| BI-E 06 | *E. sclateri* | 2022 | Bounty Islands | Proclamation Island | –47.7496 | 179.0259 | DNeasy |
| BI-E 08 | *E. sclateri* | 2022 | Bounty Islands | Proclamation Island | –47.7496 | 179.0259 | DNeasy |
| BI-E 10 | *E. sclateri* | 2022 | Bounty Islands | Proclamation Island | –47.7496 | 179.0259 | DNeasy |
| BI-E 11 | *E. sclateri* | 2022 | Bounty Islands | Proclamation Island | –47.7496 | 179.0259 | DNeasy |
| BI-E 12 | *E. sclateri* | 2022 | Bounty Islands | Proclamation Island | –47.7496 | 179.0259 | DNeasy |
| BI-E 13 | *E. sclateri* | 2022 | Bounty Islands | Proclamation Island | –47.7496 | 179.0259 | DNeasy |
| BI-E 14 | *E. sclateri* | 2022 | Bounty Islands | Proclamation Island | –47.7496 | 179.0259 | DNeasy |
| BI-E 15 | *E. sclateri* | 2022 | Bounty Islands | Proclamation Island | –47.7496 | 179.0259 | DNeasy |
| BI-E 16 | *E. sclateri* | 2022 | Bounty Islands | Proclamation Island | –47.7496 | 179.0259 | DNeasy |
| BI-E 17 | *E. sclateri* | 2022 | Bounty Islands | Proclamation Island | –47.7496 | 179.0259 | DNeasy |
| BI-E 19 | *E. sclateri* | 2022 | Bounty Islands | Proclamation Island | –47.7496 | 179.0259 | DNeasy |
| BI-E 20 | *E. sclateri* | 2022 | Bounty Islands | Proclamation Island | –47.7496 | 179.0259 | DNeasy |
| BI-E 21 | *E. sclateri* | 2022 | Bounty Islands | Proclamation Island | –47.7496 | 179.0259 | DNeasy |
| BI-E 22 | *E. sclateri* | 2022 | Bounty Islands | Proclamation Island | –47.7496 | 179.0259 | DNeasy |
| BI-E 23 | *E. sclateri* | 2022 | Bounty Islands | Proclamation Island | –47.7496 | 179.0259 | DNeasy |
| BI-E 24 | *E. sclateri* | 2022 | Bounty Islands | Proclamation Island | –47.7496 | 179.0259 | DNeasy |
| BI-E 25 | *E. sclateri* | 2022 | Bounty Islands | Proclamation Island | –47.7496 | 179.0259 | DNeasy |
| BI-E 26 | *E. sclateri* | 2022 | Bounty Islands | Proclamation Island | –47.7496 | 179.0259 | DNeasy |
| BI-E 27 | *E. sclateri* | 2022 | Bounty Islands | Proclamation Island | –47.7496 | 179.0259 | DNeasy |
| BI-E 28 | *E. sclateri* | 2022 | Bounty Islands | Proclamation Island | –47.7496 | 179.0259 | DNeasy |
| BI-E 29 | *E. sclateri* | 2022 | Bounty Islands | Proclamation Island | –47.7496 | 179.0259 | DNeasy |
| SC-E 01 | *E. sclateri* | 2022 | Antipodes Island | South Coast | –49.7063 | 178.7483 | DNeasy |
| SC-E 02 | *E. sclateri* | 2022 | Antipodes Island | South Coast | –49.7063 | 178.7483 | DNeasy |
| SC-E 03 | *E. sclateri* | 2022 | Antipodes Island | South Coast | –49.7063 | 178.7483 | DNeasy |
| SC-E 04 | *E. sclateri* | 2022 | Antipodes Island | South Coast | –49.7063 | 178.7483 | DNeasy |
| SC-E 05 | *E. sclateri* | 2022 | Antipodes Island | South Coast | –49.7063 | 178.7483 | DNeasy |
| SC-E 06 | *E. sclateri* | 2022 | Antipodes Island | South Coast | –49.7063 | 178.7483 | DNeasy |
| SC-E 07 | *E. sclateri* | 2022 | Antipodes Island | South Coast | –49.7063 | 178.7483 | DNeasy |
| SC-E 08 | *E. sclateri* | 2022 | Antipodes Island | South Coast | –49.7063 | 178.7483 | DNeasy |
| SC-E 09 | *E. sclateri* | 2022 | Antipodes Island | South Coast | –49.7063 | 178.7483 | DNeasy |
| SC-E 11 | *E. sclateri* | 2022 | Antipodes Island | South Coast | –49.7063 | 178.7483 | DNeasy |
| SC-E 12 | *E. sclateri* | 2023 | Antipodes Island | South Coast | –49.7063 | 178.7483 | DNeasy |
| SC-E 13 | *E. sclateri* | 2022 | Antipodes Island | South Coast | –49.7063 | 178.7483 | DNeasy |
| SC-E 14 | *E. sclateri* | 2022 | Antipodes Island | South Coast | –49.7063 | 178.7483 | DNeasy |
| SC-E 15 | *E. sclateri* | 2022 | Antipodes Island | South Coast | –49.7063 | 178.7483 | DNeasy |
