# Supplementary material for: Population structure of three New Zealand crested penguins identifies current conservation challenges for the Fiordland penguin/tawaki, erect-crested penguin, and eastern rockhopper penguin
Source: PLoS One. 2025 Aug 27;20(8):e0329545. doi: 10.1371/journal.pone.0329545 (PMC12385409; doi:10.1371/journal.pone.0329545)
Supplement: Attachment — Submitted filename: Marked_Pdf.pdf [file pone.0329545.s002.pdf]

PLOS ONE

Population structure of three New Zealand crested penguins identifies current conservation challenges: Fiordland penguin/tawaki, erect-crested penguin, and Eastern rockhopper penguin

--Manuscript Draft--

**Manuscript Number:**	PONE-D-24-41700
**Article Type:**	Research Article
**Full Title:**	Population structure of three New Zealand crested penguins identifies current conservation challenges: Fiordland penguin/tawaki, erect-crested penguin, and Eastern rockhopper penguin
**Short Title:**	Population structure of three New Zealand crested penguins identifies current conservation challenges
**Corresponding Author:**	Jeff White<br>Cornell University College of Veterinary Medicine<br>UNITED STATES OF AMERICA
**Keywords:**	admixture; coancestry; ddRad-seq; Eudyptes filholi; Eudyptes pachyrhynchus; Eudyptes sclateri
**Abstract:**	Identifying contemporary population structure and genetic connectivity among seabird populations is essential for developing conservation plans for threatened species. Factors like philopatry, non-breeding behavior, and oceanographic features might limit gene flow between isolated populations. Here, we characterize the population structure of three closely related crested penguin species in New Zealand: Fiordland penguins (Eudyptes pachyrhynchus; tawaki), erect-crested penguins (Eudyptes sclateri), and Eastern rockhopper penguins (Eudyptes filholi). Whereas tawaki populations appear to be stable, the sub-Antarctic erect-crested and Eastern rockhopper penguins populations have seen dramatic declines. To understand the genetic implications of these differences in population trajectories, we assessed genetic connectivity among multiple colonies using thousands of nuclear autosomal loci. Our results indicate that Tawaki are a single, genetically diverse population without colony-based structure. However, we identified two genetically distinct populations of erect-crested penguins corresponding to the Antipodes Islands and the Bounty Islands groups. The Antipodes Islands Eastern rockhopper population exhibited high levels of coancestry and low genetic diversity, consistent with population decline and limited immigration. The lack of gene flow and genetic diversity in both erect-crested and Eastern rockhopper penguins on the Antipodes Islands raises concerns and highlights the need for continued research to better inform conservation efforts. In contrast, Tawaki may be stable and increasing and conservation efforts should prioritize the protection of marine habitats and reducing fisheries mortality.
**Order of Authors:**	Jeff White
	Philip Lavretsky
	Pablo Garcia-Borboroglu
	Ursula Ellenberg
	David Houston
	Robin Long
	Thomas Mattern
	Herman L Mays Jr
	Klemens Putz
	Philip Seddon
	Kevin G McCracken
**Additional Information:**	

Powered by Editorial Manager® and ProduXion Manager® from Aries Systems Corporation

Question	Response
**Financial Disclosure**<br><br>Enter a financial disclosure statement that describes the sources of funding for the work included in this submission. Review the submission guidelines for detailed requirements. View published research articles from PLOS ONE for specific examples.<br><br>This statement is required for submission and **will appear in the published article if** the submission is accepted. Please make sure it is accurate.<br><br>**Funded studies**<br>Enter a statement with the following details:<br>• Initials of the authors who received each award<br>• Grant numbers awarded to each author<br>• The full name of each funder<br>• URL of each funder website<br>• Did the sponsors or funders play any role in the study design, data collection and analysis, decision to publish, or preparation of the manuscript?<br><br>Did you receive funding for this work?	Yes
Please add funding details.<br>as follow-up to "**Financial Disclosure**<br><br>Enter a financial disclosure statement that describes the sources of funding for the work included in this submission. Review the submission guidelines for detailed requirements. View published research articles from PLOS ONE for specific examples.<br><br>This statement is required for submission and **will appear in the published article if** the submission is accepted. Please make sure it is accurate.	1.Vontobel Foundation (Award Von_1-2022_ART) – KP, TM<br>https://www.vontobel.com/<br>2.James A. Kushlan Endowment for Waterbird Biology and Conservation at the University of Miami – KGM<br>https://news.miami.edu/as/stories/2013/09/kevin-g-mccracken-named-inaugural-kushlan-chair-in-waterbird-biology-and-conser.html<br>3.National Science Foundation (Award #2322123) – HLM<br>https://new.nsf.gov/funding<br>4.Antarctic Research Trust – KP<br>https://antarctic-research.de/wordpress2014/?page_id=1900&lang=en<br>5.Global Penguin Society – PGB<br>https://www.globalpenguinsociety.org/<br>6.Royal Naval Birdwatching Society – JW<br>https://www.rnbws.org.uk/<br>7.Birds New Zealand Research Fund (BNZRF) – JW<br>https://www.birdsnz.org.nz/research/<br>8.Shearwater Foundation – JW<br>https://shearwaterfoundation.org/<br>9.Patreon Supporters of the Tawaki Project – TM, UE

Powered by Editorial Manager® and ProduXion Manager® from Aries Systems Corporation

**Funded studies**<br>Enter a statement with the following details:<br>• Initials of the authors who received each award<br>• Grant numbers awarded to each author<br>• The full name of each funder<br>• URL of each funder website<br>• Did the sponsors or funders play any role in the study design, data collection and analysis, decision to publish, or preparation of the manuscript?<br><br>Did you receive funding for this work?"	https://www.patreon.com/TawakiProject<br><br>The funders had no role in study design, data collection and analysis, decision to publish, or preparation of the manuscript.
Please select the country of your main research funder (please select carefully as in some cases this is used in fee calculation).<br>as follow-up to "**Financial Disclosure**<br><br>Enter a financial disclosure statement that describes the sources of funding for the work included in this submission. Review the submission guidelines for detailed requirements. View published research articles from PLOS ONE for specific examples.<br><br>This statement is required for submission and **will appear in the published article if** the submission is accepted. Please make sure it is accurate.<br><br>**Funded studies**<br>Enter a statement with the following details:<br>• Initials of the authors who received each award<br>• Grant numbers awarded to each author<br>• The full name of each funder<br>• URL of each funder website<br>• Did the sponsors or funders play any role in the study design, data collection and analysis, decision to publish, or preparation of the manuscript?<br><br>Did you receive funding for this work?"	SWITZERLAND - CH
**Competing Interests**	The authors have declared that no competing interests exist.

Use the instructions below to enter a competing interest statement for this submission. On behalf of all authors, disclose any competing interests that could be perceived to bias this work—acknowledging all financial support and any other relevant financial or non-financial competing interests.<br><br>This statement is required for submission and **will appear in the published article if** the submission is accepted. Please make sure it is accurate and that any funding sources listed in your Funding Information later in the submission form are also declared in your Financial Disclosure statement.<br><br>View published research articles from PLOS ONE for specific examples.<br><br>**NO authors have competing interests**<br><br>Enter: The authors have declared that no competing interests exist.<br><br>**Authors with competing interests**<br><br>Enter competing interest details beginning with this statement:<br><br>I have read the journal's policy and the authors of this manuscript have the following competing interests: [insert competing interests here]<br><br>* typeset	
**Ethics Statement**<br><br>Enter an ethics statement for this submission. This statement is required if the study involved:<br><br>• Human participants<br>• Human specimens or tissue<br>• Vertebrate animals or cephalopods<br>• Vertebrate embryos or tissues	This project was approved by the Marshall University Office of Research Integrity's Institutional Animal Care and Use Committee (IACUC) under protocol #686, the University of Miami Institutional Animal Care and Use Committee (IACUC) under protocol #20-090, and the University of Otago's Animal Ethics Committee (#AUP D69/17). All field work and permissions were granted under the Department of Conservation (DOC) permit authorization numbers-FAU 86101-FAU and 78612-FAU.

Powered by Editorial Manager® and ProduXion Manager® from Aries Systems Corporation

• Field research<br><br>Write "N/A" if the submission does not require an ethics statement.<br><br>General guidance is provided below. Consult the submission guidelines for detailed instructions. **Make sure that all information entered here is included in the Methods section of the manuscript.**	

Powered by Editorial Manager® and ProduXion Manager® from Aries Systems Corporation

**Format for specific study types**<br><br>**Human Subject Research (involving human participants and/or tissue)**<br>• Give the name of the institutional review board or ethics committee that approved the study<br>• Include the approval number and/or a statement indicating approval of this research<br>• Indicate the form of consent obtained (written/oral) or the reason that consent was not obtained (e.g. the data were analyzed anonymously)<br><br>**Animal Research (involving vertebrate animals, embryos or tissues)**<br>• Provide the name of the Institutional Animal Care and Use Committee (IACUC) or other relevant ethics board that reviewed the study protocol, and indicate whether they approved this research or granted a formal waiver of ethical approval<br>• Include an approval number if one was obtained<br>• If the study involved non-human primates, add additional details about animal welfare and steps taken to ameliorate suffering<br>• If anesthesia, euthanasia, or any kind of animal sacrifice is part of the study, include briefly which substances and/or methods were applied<br><br>**Field Research**<br><br>Include the following details if this study involves the collection of plant, animal, or other materials from a natural setting:<br>• Field permit number<br>• Name of the institution or relevant body that granted permission	
**Data Availability**<br><br>Authors are required to make all data underlying the findings described fully available, without restriction, and from the time of publication. PLOS allows rare exceptions to address legal and ethical concerns. See the PLOS Data Policy and FAQ for detailed information.	Yes - all data are fully available without restriction

A Data Availability Statement describing where the data can be found is required at submission. Your answers to this question constitute the Data Availability Statement and **will be published in the article, if** accepted.<br><br>**Important: Stating 'data available on request** from the author' is not sufficient. If your data are only available upon request, select 'No' for the first question and explain your exceptional situation in the text box.<br><br>Do the authors confirm that all data underlying the findings described in their manuscript are fully available without restriction?	
**Describe where the data may be found in full sentences. If you are copying our sample text, replace any instances of XXX with the appropriate details.**<br><br>• If the data are **held or will be held in a public repository, include URLs,** accession numbers or DOIs. If this information will only be available after acceptance, indicate this by ticking the box below. For example: All XXX files are available from the XXX database (accession number(s) XXX, XXX).<br>• If the data are all contained **within the manuscript and/or Supporting Information files, enter the following:** All relevant data are within the manuscript and its Supporting Information files.<br>• If neither of these applies but you are able to provide **details of access elsewhere, with or without limitations,** please do so. For example:<br><br>Data cannot be shared publicly because of [XXX]. Data are available from the XXX Institutional Data Access / Ethics Committee (contact via XXX) for researchers who meet the criteria for access to confidential data.<br><br>The data underlying the results presented in the study are available from (include the name of the third party	All relevant data will be available at https://www.duckdna.org/data/ and in the NCBI SRA database.

and contact information or URL).<br>• This text is appropriate if the data are owned by a third party and authors do not have permission to share the data.<br><br>* typeset	
Additional data availability information:	Tick here if the URLs/accession numbers/DOIs will be available only after acceptance of the manuscript for publication so that we can ensure their inclusion before publication.

Powered by Editorial Manager® and ProduXion Manager® from Aries Systems Corporation

Manuscript

Click here to access/download;Manuscript;white_manuscript_PLOSone_11_S

Population structure of three New Zealand crested penguins identifies current conservation challenges: Fiordland penguin/tawaki, erect-crested penguin, and Eastern rockhopper penguin

Jeff White[1,2,#a*], Philip Lavretsky[3], Pablo Garcia Borboroglu[4,5,6], Ursula Ellenberg[4,6,7], David Houston[6,8], Robin Long[9], Thomas Mattern[4,6,10], Herman L. Mays Jr.[2], Klemens Pütz[11], Philip Seddon[10], and Kevin G. McCracken[1,12,13]

[1]Department of Biology, University of Miami, Coral Gables, Florida, United States of America

[2]Department of Biology, Marshall University, Huntington, West Virginia, United States of America

[3]Department of Biological Science, University of Texas El Paso, El Paso, Texas, United States of America

[4]Global Penguin Society, Puerto Madryn, Chubut, Argentina

[5]CESIMAR CONICET (National Research Council), Argentina

[6]The Tawaki Trust, Dunedin, New Zealand

[7]Department of Marine Sciences, University of Otago, Dunedin, New Zealand

[8]Department of Conservation, Auckland, New Zealand

[9]West Coast Penguin Trust, Hokitika, New Zealand

[10]Department of Zoology, University of Otago, Dunedin, New Zealand

[11]Antarctic Research Trust, Bremervörde, Germany

[12]Department of Marine Biology and Ecology, Rosenstiel School of Marine, Atmospheric, and Earth Science, University of Miami, Miami, Florida, United States of America

[13]Human Genetic and Genomics, University of Miami Miller School of Medicine, Miami, Florida, United States of America

[#a] Current address: Department of Public and Ecosystem Health, Cornell University College of Veterinary Medicine, Ithaca, New York, United States of America

*Corresponding author

E-mail: jeffwwhite90@gmail.com (JW)

Author Contributions

Conceptualization: JW, KGM, TM; Data Curation and Formal Analysis: JW, KGM, PL; Funding Acquisition: JW, KGM, TM, PBG, HLM, KP; Investigation: JW, TM, UE, DH, RL, KP; Methodology: JW, KGM, TM, PL, HLM; Writing (original draft): JW; Writing (review and editing): JW, PL, PGB, UE, DH, RL, TM, HLM, KP, PS, KGM

KEYWORDS: admixture, coancestry, ddRad-seq, *Eudyptes filholi*, *Eudyptes pachyrhynchus*, *Eudyptes sclateri*

Abstract

Identifying contemporary population structure and genetic connectivity among seabird populations is essential for developing conservation plans for threatened species. Factors like philopatry, non-breeding behavior, and oceanographic features might limit gene flow between isolated populations. Here, we characterize the population structure of three closely related crested penguin species in New Zealand: Fiordland penguins (*Eudyptes pachyrhynchus*; tawaki), erect-crested penguins (*Eudyptes sclateri*), and Eastern rockhopper penguins (*Eudyptes filholi*). Whereas tawaki populations appear to be stable, the sub-Antarctic erect-crested and Eastern rockhopper penguins populations have seen dramatic declines. To understand the genetic implications of these differences in population trajectories, we assessed genetic connectivity among multiple colonies using thousands of nuclear autosomal loci. Our results indicate that Tawaki are a single, genetically diverse population without colony-based structure. However, we identified two genetically distinct populations of erect-crested penguins corresponding to the Antipodes Islands and the Bounty Islands groups. The Antipodes Islands Eastern rockhopper population exhibited high levels of coancestry and low genetic diversity, consistent with population decline and limited immigration. The lack of gene flow and genetic diversity in both erect-crested and Eastern rockhopper penguins on the Antipodes Islands raises concerns and highlights the need for continued research to better inform conservation efforts. In contrast, Tawaki may be stable and increasing and conservation efforts should prioritize the protection of marine habitats and reducing fisheries mortality.

Introduction

Wildlife conservation management requires an understanding of demographic histories and population interconnectedness (i.e., gene flow), as both are essential for addressing genetic diversity declines in the face of ongoing population size changes in ever-increasingly fragmented and altered landscapes and seascapes [1-3]. Seabirds are among the most threatened groups of vertebrates worldwide, with up to 70% of seabird species currently declining [4,5]. This is thought to be primarily due to the negative impacts of invasive species, fisheries bycatch, and climate change [6]. However, these threats do not apply evenly across all groups of seabirds. For example, penguins are particularly vulnerable to the effects of climate change as well as fisheries interactions [6-9]. Therefore, understanding the ecology and evolutionary history of species through studies of population genetic structure are vital, as they reveal past demographic histories and may identify populations of conservation concern [10].

Although seabirds are generally highly mobile and capable of covering large distances at sea, most seabird species exhibit strong philopatry to breeding sites [11]. This can be an isolating mechanism that creates barriers to gene flow between populations [12], and potentially reduces a population's ability to recover from catastrophic events [13-15]. The dispersal of seabirds is also limited by hydrography [16]. In particular, frontal zones separating water masses have been identified as significant barriers to gene flow in some penguin species [17-19]. Such oceanographic barriers can reduce overlap in foraging areas among populations during both breeding and non-breeding periods. Over time, this may result in allochrony within the annual cycle as populations shift the timing of events (i.e., breeding, non-breeding dispersal) to coincide with optimal foraging conditions within their preferred foraging area [20-23]. In some cases,

allochrony and marine habitat segregation can eventually lead to speciation [24]. However, in other cases such as with king penguins (*Aptenodytes patagonicus)*, wide ranging seabird species can also show very little genetic structure [25].

Study system

Our study focuses on understanding population structure, trends in population size, and genetic connectivity of three penguin species from New Zealand. The crested penguins of New Zealand present an interesting opportunity to compare the population structure of closely related species with very different dispersal patterns and population trajectories. Specifically, the Fiordland penguin (*Eudyptes pachyrhynchus*; hereafter referred to by its te reo Māori name 'tawaki') was once thought to be in decline [26], but recent surveys have indicated that populations of this species may be stable or even increasing [27-29] and expanding in range [30]. Tracking studies have shown that during the non-breeding season, tawaki are highly mobile and cover vast distances between the Sub-Tropical Front (STF) and the Sub-Antarctic Front (SAF) to forage in sub-Antarctic waters [31]. Previous studies using single-locus mitochondrial DNA indicated that the Tawaki population has remained stable with continued gene flow over the last 1,151 years [32]. However, finer scale gene flow among individual colonies has not been assessed using modern DNA sequencing methods that incorporate many loci.

In contrast, the crested penguin species inhabiting New Zealand's Subantarctic islands have been in decline for decades. For example, Eastern rockhopper penguins (*Eudyptes filholi*) have declined by 94% on Campbell Island since the 1940s [33]. On the Antipodes Islands, the Eastern rockhopper penguin population dropped by 92% over a 22-year period [34, 35]. A recent survey of the Orde Lees colony on Antipodes Island reported a 46% decline since 2011 alone [36]. This

decline in Eastern rockhopper penguins in New Zealand is thought to be primarily a result of changing marine conditions leading to alterations in prey availability [34, 35, 37, 38].

The erect-crested penguin (*Eudyptes sclateri*) is currently found only on the Antipodes Islands and in the Bounty Islands group. Erect-crested penguins show strikingly different population trends between island groups. On the Antipodes Islands the breeding population of Erect-crested penguins has declined by 29‒42% over the last decade [35, 36]. Yet on the Bounty Islands, erect-crested penguins have remained relatively stable over the same period [39], such that the Bounty Islands now host most of the breeding population [36]. The two populations of erect-crested penguins exhibit slight breeding allochrony, with the Bounty Islands population finishing the breeding season 2-3 weeks later than that on the Antipodes Islands [40]. The populations also have differing foraging behavior during the pre-molt period. The Antipodes Islands penguins remain south of the STF during the pre-molt, while those from the Bounty Islands forage along the STF itself nearer to their breeding colonies [41]. The diverging dispersal patterns during the pre-molt period suggests that the STF influences penguin movements, thus driving the segregation between the Bounty and Antipodes Islands populations.

To understand potential genetic risks for these penguins, we explored whether population structure exists among and within tawaki, Eastern rockhopper, and erect-crested penguins. We utilized genome-wide, single nucleotide polymorphism (SNP) data to compare range-wide patterns of genetic diversity among and within these species to characterize population structure, including the degree of connectivity between intraspecific colonies. Among species, we were interested in comparing the different genetic histories of each taxon as it relates to the demography and geography of each. For tawaki we were interested in establishing the degree of

gene flow among breeding colonies and determining whether there is genetic evidence that this species indeed represents a single, unstructured population. In contrast to tawaki, in which we expected to find a high degree of genetic diversity, we expect decreased diversity among Eastern rockhopper penguin populations of the Antipodes Islands in light of their alarming declines. Finally, we investigated whether the presence of the STF, and associated higher primary productivity, influences gene flow between the Antipodes Islands and Bounty Islands populations of erect-crested penguins.

This project was approved by the Marshall University Office of Research Integrity's Institutional Animal Care and Use Committee (IACUC) under protocol #686, the University of Miami Institutional Animal Care and Use Committee (IACUC) under protocol #20-090, and the University of Otago's Animal Ethics Committee (#AUP D69/17). All field work and permissions were granted under the Department of Conservation (DOC) permit authorization numbers-FAU 86101-FAU and 78612-FAU.

Materials and Methods

Sample collection

We collected blood samples (1 mL) from the brachial vein of adult tawaki (n = 55 individuals) across five colonies during the breeding seasons (September-October) of 2017, 2018, and 2022 (Fig 1; S1 Table). These colonies were divided into four regions: South Westland (Jackson Head), Milford Sound (Harrison Cove), Doubtful Sound (East Shelter and Seymour Islands), and Foveaux Strait (Whenua Hou). The sex of all tawaki was determined in the field using key morphometric characters [42]. We then collected blood samples from erect-crested and Eastern rockhopper penguins in November-December 2022 (S1 Table). Erect-crested penguins were sampled from the north and south coasts of Antipodes Island as well as on Proclamation Island in the Bounty Islands

group (Fig 1). Eastern rockhopper penguins were sampled on the north and south sides of Antipodes Island only as they do not occur in the Bounty Islands (Fig 1). We determined sex for erect-crested and Eastern rockhopper penguins in the field following Davis et al. [43] and Warham [44], respectively. All samples collected in 2017 and 2018 were stored in the field in 70% ethanol, whereas those collected in 2022 were stored in a blood lysis buffer [45]. Upon returning to the lab, all samples were frozen and stored at –80°C.

Fig 1. Map of all sampling locations. All sampling occurred in southern New Zealand during the breeding seasons of 2017, 2018, and 2022. Colored points represent sampling locations for tawaki (purple, green, orange, yellow; $n = 55$), erect-crested penguins (red, blue; $n = 54$), and Eastern rockhopper penguins (blue; $n = 17$).

DNA extraction and ddRAD-Seq library preparation

We extracted DNA from each blood sample either using a DNeasy Blood and Tissue kit following the standard manufacturer protocols (Qiagen, Valencia, CA, USA) or with phenol-chloroform-isoamyl alcohol extractions. We then performed partial genome sequencing via double-digest restriction-associated DNA (ddRAD) sequencing methods following protocols of DaCosta and Sorenson [46], but with size-selection following Hernández et al. [47]. In short, genomic DNA was first enzymatically digested using SbfI and EcoRI restriction enzymes followed by adapter ligation with Illumina TrueSeq compatible reagents and barcodes for demultiplexing. Next, we conducted double-side bead-based size selection of adapter-ligated DNA fragments [47]. Finally, clean libraries were multiplexed in equimolar amounts and sent out for sequencing on an Illumina HiSeq X using single-end 150 bp chemistry at Novogene Ltd. (Sacramento, CA, USA).

Bioinformatics of ddRad-Seq data

All raw Illumina reads were first demultiplexed using the ddRADparser.py python script of the DaCosta and Sorenson (http://github.com/BURAD-seq/ddRad-seq-Pipeline) pipeline [46]. Next, all reads were cleaned using Trimmomatic v0.36v [48] before being aligned to the macaroni penguin (*Eudyptes chrysolophus)* reference genome (https://www.ncbi.nlm.nih.gov/datasets/genome/GCA_010084205.1/) using Burrows Wheeler Aligner v.0.7.1.7 [49]. Samples were then sorted and indexed in Samtools v.1.6 [50] and combined and genotyped using bcftools v.1.6 (as part of the SAMtools package) "mpileup" and "call" functions with the following parameters "-c –A -Q 30 -q 30," which set a base pair and overall sequence PHRED score of ≥30 to ensure that only high-quality sequences are retained. Note all steps through genotyping were automated using in-house Python scripts (https://github.com/jonmohl.PopGen; [51]). Next, we used VCFtools v. 0.115 [52] to further filter VCF files for any base-pair missing >20% of samples, which also required a minimum base-pair sequencing depth coverage of 5X (i.e., 10X per genotype) and quality per base PHRED scores of ≥30 to be retained.

Population structure

Population structure analyses were based on autosomal ddRAD-seq biallelic single nucleotide polymorphisms (SNPs) only. We used PLINK v.1.90 [53] to ensure that singletons and any SNP missing >20% of data across samples were excluded in each dataset. Additionally, we identified independent SNPs by conducting pairwise linkage disequilibrium (LD) tests across ddRAD-seq autosomal SNPs (--indep-pairwise 2 1 0.5) in which 1 of 2 linked SNPs are randomly excluded if we obtained an LD correlation factor (r^2) > 0.5. All analyses were done without *a priori* information on population or species identity. Note these steps were done for each separate

analysis that included (1) All penguins, (2) tawaki only, (3) erect-crested penguins only, and (4) Eastern rockhopper penguins only.

Next, we used the PCA function in PLINK to perform a principal component analysis (PCA). ADMIXTURE v.1.3 [54, 55] was used to attain maximum likelihood estimates of population assignments for each individual, with datasets formatted for the ADMIXTURE analysis using PLINK v.1.90, and following steps outlined in Alexander et al. [54]. We ran each ADMIXTURE analysis with a 10-fold cross validation, incorporating a quasi-Newton algorithm to accelerate convergence [56]. Each analysis used a block relaxation algorithm for point estimation and terminated once the change in the log-likelihood of the point estimations increased by <0.0001. Each analysis was run for K population values with standard-errors derived from 100 bootstrap replicates per each value of K. We ran $K = 1–10$ for the all-species analyses and 1–5 values for species specific analyses. The optimum K in each analysis was based on cross-validation errors per K value; however, we examined additional values of K to test for further structural resolution across analyses. Finally, we also visualized coancestry assignments using fineRADstructure v.0.3 [57, 58]. This method infers a matrix of coancestry coefficients based on the distribution of identical or nearest neighbor haplotypes among samples. Coancestry at each locus is divided equally among all individuals with identical haplotypes, or in the case of a unique allele, with the nearest neighbor haplotype [58]. Rare haplotypes, as characterized by rare SNPs, are on average of more recent origin [59] and contribute the most to the coancestry index. This then provides a measure that highlights recent coancestry. We completed a burn-in of 100,000 iterations, followed by 100,000 Markov chain Monte Carlo iterations. Finally, we constructed the phylogenetic tree using the software's default parameters.

Summary statistics

Summary statistics including pair-wise estimates of relative genetic divergence (pairwise Φ_{ST}) and average per group nucleotide diversity (π) were calculated among all three species in VCFtools. We also calculated π and Tajima's D among locations within the tawaki and erect-crested penguin samples. For the erect-crested penguins, we were interested in the connectivity of the Antipodes Islands and Bounty Islands groups and therefore calculated these statistics between the two island groups as well.

Results

Population structure

A total of 436,292 base-pairs (bps) met threshold criteria, with average individual sequencing depth of 71X (range = 7–113X). For the populations structure analyses, we had a total of 19,974 (of 21,513 and 97% of alleles present), 8,953 (of 9,991 and 96% of alleles present), 5,018 (of 5,760 and 99% of alleles present), and 5,213 (of 7,173 and 99% of alleles present) SNPs for all-species combined, erect-crested penguin, tawaki, and Eastern rockhopper analyses, respectively. Concordant with the ADMIXTURE analysis, we found $K = 3$ populations to be the optimum model (Table 1). Furthermore, the same three genetic clusters were recovered in both the PCA and fineRADstructure coancestry analysis (Figs 2 and 3). No subpopulation structure was found among Tawaki populations for which the coancestry assignments (Fig 3) and ADMIXTURE analyses found an optimum model of $K = 1$ within this species (Table 1). Conversely, both the PCA (Fig 4) and ADMIXTURE assignment probabilities (Fig 5) revealed two distinct genetic clusters among erect-crested penguins corresponding to the Antipodes and Bounty populations, including putative hybrids between these two populations.

Fig 2. Principal components analysis (PCA) of all ddRAD-seq data. Tawaki (circle), erect-crested penguins (square), and Eastern rockhopper penguins (asterisk) colors denote the region in which the samples were collected. The first two principal components PC1 and PC2 represent 60.4% of the variance and separate the samples into three distinct genetic clusters.

Fig 3. fineRADstructure heat map. Heat map resulting from the population assignment analysis performed using fineRADstructure on all ddRAD-Seq data combined. Tick marks represent individual samples. The coancestry matrix shows the pairwise genetic similarity between individuals. Inferred p[illegible]lations are indicated by the dendrogram on the right. Overall, tawaki exhibit the lowest degree of coancestry whereas the Eastern rockhopper penguins exhibit very high coancestry.

Fig 4. Principal components analysis (PCA) for Erect-crested Penguins. All ddRAD-seq data for erect-crested penguins from the Anchorage Bay (circle), South Coast (triangle), and Proclamation Island (square) colonies. The first two principal components PC1 and PC2 represent 20.8% of the variance and separate the samples into two distinct genetic clusters: Antipodes Island (blue) and Bounty Islands (red). Note six individuals that fall outside of the main clusters.

Figure 5. ADMIXTURE plot for Erect-crested Penguins. ADMIXTURE plot from ddRAD-Seq data from erect-crested penguins showing assignments to population 1 or 2 based on $K = 2$. Six individuals including three from the Bounty Islands and three from the Antipodes Islands and falling outside the clusters on the PCA appear as 'hybrids' between the two groups.

Table 1. Cross validation (CV) and cluster assignment (*K*).

Group	CV	K
All samples	0.37658	1
	0.24999	2
	**0.17026**	**3**
	0.17818	4
	0.18734	5
	0.19054	6
	0.1953	7
	0.20426	8
	0.21649	9
	0.21921	10
Tawaki	**0.44067**	**1**
	0.47012	2
	0.51374	3
	0.54956	4
	0.61122	5
Erect-crested Penguin	**0.41608**	**1**
	0.45379	2
	0.49941	3
	0.54918	4
	0.61152	5
Eastern Rockhopper Penguin	**0.69060**	**1**
	0.92759	2
	1.26146	3
	1.50218	4
	1.48068	5

Cross validation (CV) and cluster assignment (*K*) for ddRAD-Seq data of all samples pooled as well as each species calculated individually. The optimal *K* assignment is presented in bold for each group.

Summary statistics

Pairwise Φ_{ST} revealed the highest differentiation between tawaki and Eastern rockhopper penguins (Φ_{ST} = 0.2778; Table 2) and lower between erect-crested penguins and tawaki (Φ_{ST} = 0.1696; Table 2) and Eastern rockhopper penguins (Φ_{ST} = 0.1621; Table 2), respectively.

Pairwise Φ_{ST} was lowest between the two island groups of erect-crested penguins (Φ_{ST} = 0.0089). Next, among the three penguin species, we recovered the lowest degree of coancestry among tawaki individuals, followed by erect-crested penguin, and with Eastern rockhopper penguins exhibiting the highest degree of coancestry, including a mated pair that are likely full siblings (Fig 5). This high degree of coancestry suggests a limited gene pool likely due to historical population declines (Table 2). Calculating Tajima's D revealed a positive and higher value in tawaki (D = 0.0880) than in either the erect-crested (D = –0.3067) or Eastern rockhopper penguins (D = –0.4383; Table 2). Tajima's D was higher in the Antipodes Islands (D = –0.1566) versus the Bounty Islands (D = –0.3240) populations of erect-crested penguins (Table 2).

Table 2. Summary statistics for ddRAD-Seq data.

Group	π	Tajima's D	Φ_{ST}
Tawaki	0.0021	0.0880	–
Erect-crested penguin (all)	0.0019	–0.3067	–
Erect-crested penguin (Antipodes)	0.0022	–0.1566	–
Erect-crested penguin (Bounty)	0.0021	–0.3240	–
Eastern Rockhopper	0.0023	–0.4383	–
Tawaki vs. Erect-crested penguin	–	–	0.1696
Tawaki vs. Erect-crested penguin (Antipodes)	–	–	0.2216
Tawaki vs. Erect-crested penguin (Bounty)	–	–	0.2052
Tawaki vs. Eastern Rockhopper	–	–	0.2778
Erect-crested penguin Antipodes vs. Bounty	–	–	0.0089
Erect-crested penguin vs. Eastern Rockhopper	–	–	0.1621
Erect-crested penguin (Antipodes) vs. Eastern Rockhopper	–	–	0.2216
Erect-crested penguin (Bounty) vs. Eastern Rockhopper	–	–	0.1579

Summary statistics for ddRAD-Seq data collected on tawaki, erect-crested penguin, and Eastern rockhopper penguins including pairwise Φ_{ST}, nucleotide diversity (π), and Tajima's D.

Discussion

Tawaki represent a panmictic population

Tawaki are known to be philopatric [60] and both socially and reproductively monogamous [61], which is expected to result in population structure [11, 17, 18]. However, our population structure analyses confirm that tawaki represent a single panmictic population (Table 3). This could potentially be explained by lower philopatry than expected or recent population expansions homogenizing genetic diversity and masking underlying population structure [62, 63]. However, Cole et al. [32] analyzed cytochrome c oxidase (COI) and the mitochondrial control region (CR) of tawaki and found no evidence of population structure alongside consistent gene flow over the last 1,151 years. Therefore, masking of population structure by recent population expansions is unlikely in tawaki. Our estimates of nuclear genetic diversity support these prior findings of mitochondrial DNA diversity and, together, provide a clearer view of the interconnectedness of tawaki colonies across their range (Fig 5). Although tawaki may have undergone range reductions since the arrival of early Polynesians, the Fiordland region in southwestern New Zealand has likely provided a crucial refugium harboring a large population with high levels of genetic diversity [32]. Therefore, continued monitoring and management of this core region of their range will be essential for the conservation of tawaki.

Table 3. Pairwise Φ_{ST} for ddRAD-Seq data collected on tawaki.

Group	Φ_{ST}
Doubtful Sound vs. Foveaux Strait	–0.0029
Doubtful Sound vs. Milford Sound	–0.0014
Doubtful Sound vs. South Westland	–0.0015
Milford Sound vs. Foveaux Strait	–0.0019

Milford Sound vs. South Westland	–0.0002
Foveaux Strait vs. South Westland	–0.0009

Pairwise Φ_{ST} for ddRAD-Seq data collected on tawaki compared across regions of origin across the breeding range indicate little population structure across the breeding range of tawaki.

Conservation concerns for the Eastern rockhopper and erect-crested penguins

Overall, our data corroborate a concerning trend for the Eastern rockhopper and erect-crested penguins. Both species have been experiencing significant declines on the Antipodes Islands over the last several decades, albeit at different rates [35, 36]. The sharp decline in Eastern rockhopper penguins on the Antipodes Islands has implications for the persistence of the species in the region. Our results suggest that there is a very high degree of coancestry among the Eastern rockhopper penguins on Antipodes Island. In fact, one breeding pair we sampled appears to be full siblings (Fig 5). Population bottlenecks are often cited to explain high levels of coancestry within a population [64-66]. However, the reduction in population size and genetic diversity following a bottleneck would be expected to rebound with even modest levels of immigration [67-69]. For example, king penguins on Macquarie Island recovered pre-bottleneck levels of genetic diversity within 80 years of a halt in human exploitation [70]. We suggest that the low level of genetic diversity in this subset of the Eastern rockhopper population is indicative of a sharp decline in the breeding population on the Antipodes Islands leaving the remaining individuals fewer options for outcrossing. This coincides with the rise of global temperatures over the 20th century as has been previously implicated in the decline of Eastern rockhoppers in the region [37, 38]. Our analysis also suggests that there could be little gene flow to the Antipodes Islands from other colonies in the region such as those on Auckland or Campbell Islands, further reducing chances of genetic recovery. Overall, the Eastern rockhoppers on the Antipodes Islands may be experiencing a contemporary bottleneck due to these sharp declines.

While the exact causes of this decline are not fully known, changing marine conditions and shifting prey abundance and distribution are likely contributing factors [35]. Further research should be undertaken to characterize the degree of coancestry within and between the Antipodes, Campbell, and Auckland Island populations to better understand the magnitude of our findings for the species as a whole in the region.

The erect-crested penguin presents a unique case among New Zealand penguins. It is found only on the Antipodes and Bounty Island groups, yet the population trajectories are different in each location. Most of the population (62.9%) is now situated in the Bounty Islands as the Antipodes Islands population continues to decline (Mattern et al., in prep.). However, there seems to have been little decline in genetic diversity in this species through time [32]. As for the Eastern rockhopper penguins, the cause of the decline on the Antipodes Islands is thought to be due primarily to changing marine conditions and, thus, prey abundance [35]. Our results suggest that erect-crested penguins comprise two distinct subpopulations corresponding to the Antipodes Islands and Bounty Islands groups. The small proportion of the overall Bounty Islands population that had Antipodes Islands ancestry suggests that the more stable population of the Bounty Islands is not being significantly augmented by immigration from the Antipodes Islands (Figs 3 and 4; Table 2). While we cannot conclusively rule out other factors, the available evidence suggests that the decline in the Antipodes Islands population is most likely driven by ongoing environmental stressors rather than significant emigration.

We posit that the apparent population structure is likely at least partially explained by allochrony in their breeding cycles that results in each not significantly overlapping while at sea, at least during the pre-molt period [41]. In short, the STF sits just to the north of the Bounty Islands bringing high primary productivity to the region, likely negating the need for foraging penguins

to travel long distances in search of sufficient prey. Effectively utilizing different marine habitats (Bounty Islands = subtropical, Antipodes Islands = subantarctic) appears to contribute to a delayed breeding cycle on the Bounty versus Antipodes Islands. The Snares penguin (*Eudyptes robustus*) also exhibits an asynchronous annual cycle with a 2–5 week delay in breeding onset between colonies on the main Snares group and the Western Chain [71-73]. Yet there is no oceanic front that could help explain this difference. Other underlying factors must be influencing these behavioral differences in Snares penguins, and therefore should not be ruled out in understanding similar patterns in erect-crested penguins.

The presence of frontal zones has been identified as a barrier to gene flow in other penguin species [17, 19]. However, in the case of erect-crested penguins, the STF is not between the two island groups. Yet the differences in primary productivity fueled by the STF is likely an important factor resulting in behavioral differences which lead to isolation between these two populations. Most crested penguin species travel south during the pre-molt period [31, 74, 75], and the erect-crested penguins on the Antipodes Islands follow this pattern [41]. However, on the Bounty Islands, erect-crested penguins have shorter foraging ranges and travel generally north towards the STF, bucking this trend [41]. It could be that the presence of highly productive waters along the STF so close to their breeding islands prevents typical dispersal patterns in erect-crested penguins from the Bounty Islands, while those on the Antipodes Islands have retained the ancestral routines to travel south (Mattern et al., in prep.).

Conclusion

Our study expands the understanding of population structure and genetic connectivity for three species of crested penguins in New Zealand. Although closely related, each species exhibits

unique ecology and population trends. Our results support previous findings that tawaki represent a single panmictic population across their range and that there is no significant structure among colonies or regions. Therefore, conservation efforts should continue to prioritize investigating the highly plastic foraging ecology of this species and the importance of the Fiordland colonies to inform proactive conservation management plans. In contrast, our results show that erect-crested penguins should be treated as two distinct populations corresponding to the Antipodes Islands and the Bounty Islands. There is likely some limited gene flow from the Antipodes Islands to the Bounty Islands, but our data do not suggest that the reverse occurs. Eastern rockhopper penguins on Antipodes Island have very limited genetic diversity due to the continued decline in their population and little gene flow from other island groups. We encourage continued effort to identify the root causes of the continued decline of both species on the Antipodes Islands.

Acknowledgements

We would like to thank Andrea Faris, Jacob Barrett, Daniel Crook, Holly Langley (Southern Discoveries), and Rosco Gaudin (Rosco’s Kayaks) for providing logistic support to access colonies in Milford Sound. Access to Doubtful Sound colonies was made possible by Richard ‘Abbo’ and Mandy Abernathy’s invaluable support (Fiordland Expeditions). We would also like to say a huge thank you to skipper Steve Kafka and the entire crew of the research vessel *Evohe* for providing transport and support into the Subantarctic islands. A massive thanks to the many field and lab assistants that have helped on this project including Blake Hornblow, Myrene Otis, Lindsey Chan, Briana Gibbs, Hannah Mattern, and Bianca Keys. Special thanks to the Department of Conservation. Particularly Sharon Trainor, Janice Kevern, and Rhuaridh Hannan

for quarantine help, Ros Cole and Joseph Roberts for help with Entry Permits, and Graeme Taylor, Igor Debski, Johannes Fischer, Kris Ramm, Hendrik Schultz, and Sanjay Thakur for their help obtaining the necessary Wildlife Authority.

Ethics Statement

This project was approved by the Marshall University Office of Research Integrity’s Institutional Animal Care and Use Committee (IACUC) under protocol #686, the University of Miami Institutional Animal Care and Use Committee (IACUC) under protocol #20-090, and the University of Otago’s Animal Ethics Committee (#AUP D69/17). All field work and permissions were granted under the Department of Conservation (DOC) permit authorization numbers-FAU 86101-FAU and 78612-FAU.

References

1. Genovart M, Oro D, Juste J, Bertorelle G. What genetics tell us about the conservation of the critically endangered Balearic Shearwater? Biological Conservation. 2007 Jun 1;137(2):283-93. https://doi.org/10.1016/j.biocon.2007.02.016

2. Taylor SA, Friesen VL. Use of molecular genetics for understanding seabird evolution, ecology and conservation. Marine Ecology Progress Series. 2012 Apr 11;451:285-304. https://doi.org/10.3354/meps09694

3. Ramírez O, Gómez-Díaz E, Olalde I, Illera JC, Rando JC, González-Solís J, et al. Population connectivity buffers genetic diversity loss in a seabird. Frontiers in Zoology. 2013 Dec;10:1-5. https://doi.org/10.1186/1742-9994-10-28

4. North American Bird Conservation Initiative: NABCI. The State of the Birds, United States of America, 2022. 2022. Published report. NABCI. https://www.stateofthebirds.org/2022

5. Young L, VanderWerf E, editors. Conservation of marine birds. Academic Press; 2022 Sep 27.

6. Días MP, Martin R, Pearmain EJ, Burfield IJ, Small C, Phillips RA, et al. Threats to seabirds: a global assessment. Biological Conservation. 2019 Sep 1;237:525-37. https://doi.org/10.1016/j.biocon.2019.06.033

7. Trathan PN, García-Borboroglu P, Boersma D, Bost CA, Crawford RJ, Crossin GT, et al. Pollution, habitat loss, fishing, and climate change as critical threats to penguins. Conservation Biology. 2015 Feb;29(1):31-41. https://doi.org/10.1111/cobi.12349

8. Crawford R, Ellenberg U, Frere E, Hagen C, Baird K, Brewin P, et al. Tangled and drowned: a global review of penguin bycatch in fisheries. Endangered Species Research. 2017 Nov 30;34:373-96. https://doi.org/10.3354/esr00869

9. Gimeno M, Giménez J, Chiaradia A, Davis LS, Seddon PJ, Ropert-Coudert Y, et al. Climate and human stressors on global penguin hotspots: Current assessments for future conservation. Global change biology. 2024 Jan;30(1):e17143. https://doi.org/10.1111/gcb.17143

10. Duminil J, Fineschi S, Hampe A, Jordano P, Salvini D, Vendramin GG, et al. Can population genetic structure be predicted from life-history traits? The American Naturalist. 2007 May;169(5):662-72. https://doi.org/10.1086/513490

11. Coulson JC. A review of philopatry in seabirds and comparisons with other waterbird species. Waterbirds. 2016 Sep;39(3):229-40. https://doi.org/10.1675/063.039.0302

12. Milot E, Weimerskirch H, Bernatchez L. The seabird paradox: dispersal, genetic structure and population dynamics in a highly mobile, but philopatric albatross species. Molecular Ecology. 2008 Apr;17(7):1658-73. https://doi.org/10.1111/j.1365-294X.2008.03700.x

13. Welch AJ, Fleischer RC, James HF, Wiley AE, Ostrom PH, Adams J, et al. Population divergence and gene flow in an endangered and highly mobile seabird. Heredity. 2012 Jul;109(1):19-28. https://doi.org/10.1038/hdy.2012.7

14. Cimino MA, Lynch HJ, Saba VS, Oliver MJ. Projected asymmetric response of Adélie penguins to Antarctic climate change. Scientific reports. 2016 Jun 29;6(1):28785. https://doi.org/10.1038/srep28785

15. Cristofari R, Plaza P, Fernández CE, Trucchi E, Gouin N, Le Bohec C, et al. Unexpected population fragmentation in an endangered seabird: the case of the Peruvian diving-

petrel. Scientific Reports. 2019 Feb 14;9(1):2021. https://doi.org/10.1038/s41598-019-38682-9

16. Munro KJ, Burg TM. A review of historical and contemporary processes affecting population genetic structure of Southern Ocean seabirds. Emu-Austral Ornithology. 2017 Jan 2;117(1):4-18. https://doi.org/10.1080/01584197.2016.1271988

17. Clucas GV, Younger JL, Kao D, Emmerson L, Southwell C, Wienecke B, et al. Comparative population genomics reveals key barriers to dispersal in Southern Ocean penguins. Molecular Ecology. 2018 Dec;27(23):4680-97. https://doi.org/10.1111/mec.14896

18. Frugone MJ, Lowther A, Noll D, Ramos B, Pistorius P, Dantas GP, et al. Contrasting phylogeographic pattern among Eudyptes penguins around the Southern Ocean. Scientific Reports. 2018 Nov 30;8(1):17481. https://doi.org/10.1038/s41598-018-35975-3

19. Mays Jr HL, Oehler DA, Morrison KW, Morales AE, Lycans A, Perdue J, et al. Phylogeography, population structure, and species delimitation in rockhopper penguins (Eudyptes chrysocome and Eudyptes moseleyi). Journal of Heredity. 2019 Oct;110(7):801-17. https://doi.org/10.1093/jhered/esz051

20. Friesen VL, Burg TM, McCoy KD. Mechanisms of population differentiation in seabirds. Molecular Ecology. 2007 May;16(9):1765-85. https://doi.org/10.1111/j.1365-294X.2006.03197.x

21. Friesen VL, Smith AL, Gómez-Díaz E, Bolton M, Furness RW, González-Solís J, et al. Sympatric speciation by allochrony in a seabird. Proceedings of the National Academy of Sciences. 2007 Nov 20;104(47):18589-94. https://doi.org/10.1073/pnas.0700446104

22. Taylor RS, Bolton M, Beard A, Birt T, Deane-Coe P, Raine AF, et al. Cryptic species and independent origins of allochronic populations within a seabird species complex

(Hydrobates spp.). Molecular Phylogenetics and Evolution. 2019 Oct 1;139:106552. https://doi.org/10.1016/j.ympev.2019.106552

23. Medrano F, Militão T, Gomes I, Sardà-Serra M, de la Fuente M, Dinis HA, et al. Phenological divergence, population connectivity and ecological differentiation in two allochronic seabird populations. Frontiers in Marine Science. 2022 Nov 4;9:975716. https://doi.org/10.3389/fmars.2022.975716

24. Green CP, Ratcliffe N, Mattern T, Thompson D, Lea MA, Wotherspoon S, et al. The role of allochrony in influencing interspecific differences in foraging distribution during the non-breeding season between two congeneric crested penguin species. PLoS One. 2022 Feb 9;17(2):e0262901. https://doi.org/10.1371/journal.pone.0262901

25. Clucas GV, Younger JL, Kao D, Rogers AD, Handley J, Miller GD, et al. Dispersal in the sub-Antarctic: king penguins show remarkably little population genetic differentiation across their range. BMC Evolutionary Biology. 2016 Dec;16:1-4. https://doi.org/ 10.1186/s12862-016-0784-z

26. Otley H, Edmonds H, Hiscock J, Newton G, Tansell J, van Klink P, et al. Assessing the population trend and threats to New Zealand's Fiordland crested penguin using counting and demographic modelling approaches. New Zealand Journal of Ecology. 2018 Jan 1;42(2):125-36. https://doi.org/10.20417/nzjecol.42.15

27. Long R, Litchwark S. A survey of Fiordland crested penguins/tawaki (Eudyptes pachyrhynchus) from Cascade River to Martins Bay, South Westland, New Zealand, 2014. Notornis. 2017 Jan 1;64(4):206-10.

28. Long R, Litchwark S. A survey of Fiordland crested penguins (Eudyptes pachyrhynchus): northeast Stewart Island/Rakiura, New Zealand, September 2019. Notornis. 2021 Jan 1;68:183-7.

29. Mattern T, Long R. Survey and population size estimate of Fiordland penguin/tawaki (Eudyptes pachyrhynchus) in Milford Sound/Piopiotahi, New Zealand. Notornis. 2017 Jan 1;64(2):97-101.

30. Young M, Pullar C, McKinlay B. Breeding attempts by Fiordland crested penguins/tawaki (Eudyptes pachyrhynchus) on the Otago Coast. Notornis. 2015 Jan 1;62:102-4.

31. Mattern T, Pütz K, Garcia-Borboroglu P, Ellenberg U, Houston DM, Long R, et al. Marathon penguins–Reasons and consequences of long-range dispersal in Fiordland penguins/Tawaki during the pre-moult period. PLoS One. 2018 Aug 29;13(8):e0198688. https://doi.org/10.1371/journal.pone.0198688

32. Cole TL, Rawlence NJ, Dussex N, Ellenberg U, Houston DM, Mattern T, et al. Ancient DNA of crested penguins: Testing for temporal genetic shifts in the world's most diverse penguin clade. Molecular phylogenetics and evolution. 2019 Feb 1;131:72-9. https://doi.org/10.1016/j.ympev.2018.10.025

33. Cunningham DM, Moors PJ. The decline of rockhopper penguins Eudyptes chrysocome at Campbell Island, Southern Ocean and the influence of rising sea temperatures. Emu. 1994;94(1):27-36. https://doi.org/10.1071/mu9940027

34. Tennyson A, Taylor R, Taylor G, Imber M, Greene T. Unusual bird records from the Antipodes Islands in 1978-1995, with a summary of other species recorded at the island group. Notornis. 2002;49(4):241-5.

35. Hiscock JA, Chilvers BL. Declining eastern rockhopper (Eudyptes filholi) and erect-crested (E. sclateri) penguins on the Antipodes Islands, New Zealand. New Zealand Journal of Ecology. 2014 Jan 1:124-31.

36. Mattern T. The Tawaki Project - Bounty-Antipodes Expedition 2022 - 8 November - 21 December. 2023. Unpublished report, Tawaki Project, Dunedin. https://doi.org/10.13140/RG.2.2.16148.78722/1

37. Hilton GM, Thompson DR, Sagar PM, Cuthbert RJ, Cherel Y, Bury SJ. A stable isotopic investigation into the causes of decline in a sub-Antarctic predator, the rockhopper penguin Eudyptes chrysocome. Global Change Biology. 2006 Apr;12(4):611-25. https://doi:10.1111/j.1365-2486.2006.01130.x

38. Morrison KW, Battley PF, Sagar PM, Thompson DR. Population dynamics of Eastern rockhopper penguins on Campbell Island in relation to sea surface temperature 1942–2012: current warming hiatus pauses a long-term decline. Polar Biology. 2015 Feb;38:163-77. https://doi:10.1007/s00300-014-1575-x

39. Mattern T, Rexer-Huber K, Parker G, Amey J, Green CP, Tennyson AJ, et al. Erect-crested penguins on the Bounty Islands: population size and trends determined from ground counts and drone surveys. Notornis. 2021 Jan1;68(1):37-50.

40. McClelland P, Imber M, Tennyson A, Taylor G, Grant A, Greene T, et al. Antipodes Island expedition, October–November 1995. Unpublished report, Southland Conservancy, Department of Conservation, Invercargill. 2001.

41. Mattern T. The Tawaki Project - Bounty-Antipodes Expedition 2023/24 - 20 November 2023 - 2 February 2024. 2024. Unpublished report, Tawaki Project, Dunedin. https://doi.org/10.13140/RG.2.2.32269.14568

42. White J, Mattern T, Ellenberg U, Garcia-Borboroglu P, Houston DM, Seddon PJ, et al. Field sexing techniques for Fiordland crested penguins (tawaki; Eudyptes pachyrhynchus). Notornis. 2021 Jan 1;68:188-93.

43. Davis LS, Renner M, Houston D, Zhu L, Finkler W, Mattern T. The breeding biology of erect-crested penguins, Eudyptes sclateri: Hormones, behavior, obligate brood reduction and conservation. Plos one. 2022 Oct 12;17(10):e0275106. https://doi.org/10.1371/journal.pone.0275106

44. Warham J. Breeding seasons and sexual dimorphism in Rockhopper Penguins. The Auk. 1972 Jan 1;89(1):86-105. https://doi.org/10.2307/4084061

45. Longmire JL, Lewis AK, Brown NC, Buckingham JM, Clark LM, Jones MD, et al. Isolation and molecular characterization of a highly polymorphic centromeric tandem repeat in the family Falconidae. Genomics. 1988 Jan 1;2(1):14-24. https://doi.org/10.1016/0888-7543(88)90104-8

46. DaCosta JM, Sorenson MD. Amplification biases and consistent recovery of loci in a double-digest RAD-seq protocol. PloS one. 2014 Sep 4;9(9):e106713. https://doi.org/10.1371/journal.pone.0106713

47. Hernández F, Brown JI, Kaminski M, Harvey MG, Lavretsky P. Genomic evidence for rare hybridization and large demographic changes in the evolutionary histories of four North American dove species. Animals. 2021 Sep 13;11(9):2677. https://doi.org/10.3390/ani11092677

48. Bolger AM, Lohse M, Usadel B. Trimmomatic: a flexible trimmer for Illumina sequence data. Bioinformatics. 2014 Aug 1;30(15):2114-20. https://doi.org/10.1093/bioinformatics/btu170

49. Li H, Durbin R. Fast and accurate short read alignment with Burrows–Wheeler transform. bioinformatics. 2009 Jul 15;25(14):1754-60. https://doi.org/10.1093/bioinformatics/btp324

50. Li H, Handsaker B, Wysoker A, Fennell T, Ruan J, Homer N, et al. The sequence alignment/map format and SAMtools. bioinformatics. 2009 Aug 15;25(16):2078-9. https://doi.org/10.1093/bioinformatics/btp352

51. Lavretsky P, McInerney NR, Mohl JE, Brown JI, James HF, McCracken KG, et al. Assessing changes in genomic divergence following a century of human-mediated secondary contact among wild and captive-bred ducks. Molecular Ecology. 2020 Feb;29(3):578-95. https://doi.org/10.1111/mec.15343

52. Danecek P, Auton A, Abecasis G, Albers CA, Banks E, DePristo MA, et al. The variant call format and VCFtools, Bioinformatics. 2011 August;27(15):2156–2158. https://doi.org/10.1093/bioinformatics/btr330

53. Purcell S, Neale B, Todd-Brown K, Thomas L, Ferreira MAR, Bender D, et al. PLINK: a toolset for whole-genome association and population-based linkage analysis. American Journal of Human Genetics. 2007 Sep;81:559-575. https://doi.org/10.1086/519795

54. Alexander DH, Lange K. Enhancements to the ADMIXTURE algorithm for individual ancestry estimation. BMC bioinformatics. 2011 Dec;12:1-6.

55. Alexander DH, Novembre J, Lange K. Fast model-based estimation of ancestry in unrelated individuals. Genome research. 2009 Sep 1;19(9):1655-64. https://doi.org/10.1101/gr.094052.109

56. Zhou H, Alexander D, Lange K. A quasi-Newton acceleration for high-dimensional optimization algorithms. Statistics and computing. 2011 Apr;21:261-73. https://doi.org/10.1007/s11222-009-9166-3

57. Lawson DJ, Hellenthal G, Myers S, Falush D. Inference of population structure using dense haplotype data. PLoS genetics. 2012 Jan 26;8(1):e1002453. https://doi.org/10.1371/journal.pgen.1002453

58. Malinsky M, Trucchi E, Lawson DJ, Falush D. RADpainter and fineRADstructure: population inference from RADseq data. Molecular biology and evolution. 2018 May 1;35(5):1284-90. https://doi.org/10.1093/molbev/msy023

59. Kimura M, Ohta T. The age of a neutral mutant persisting in a finite population. Genetics. 1973 Sep 1;75(1):199-212.

60. Otley H, Tansell J, Scofield P. A comprehensive demographic assessment of the endangered Fiordland crested penguin Eudyptes pachyrhynchus. New Zealand Journal of Zoology. 2017 Apr 3;44(2):144-62. https://doi.org/10.1080/03014223.2017.1284135

61. McLean IG, Kayes SD, Murie JO, Davis LS, Lambert DM. Genetic monogamy mirrors social monogamy in the Fiordland crested penguin. New Zealand Journal of Zoology. 2000 Jan 1;27(4):311-6. https://doi.org/10.1080/03014223.2000.9518240

62. Hagen SB, Kopatz A, Aspi J, Kojola I, Eiken HG. Evidence of rapid change in genetic structure and diversity during range expansion in a recovering large terrestrial carnivore. Proceedings of the Royal Society B: Biological Sciences. 2015 May 22;282(1807):20150092. https://doi.org/10.1098/rspb.2015.0092

63. Haase M, Höltje H, Blahy B, Bridge D, Henne E, Johansson US, et al. Shallow genetic population structure in an expanding migratory bird with high breeding site fidelity, the

Western Eurasian Crane Grus grus grus. Journal of Ornithology. 2019 Oct;160:965-72. https://doi.org/10.1007/s10336-019-01688-1

64. Glenn TC, Stephan W, Braun MJ. Effects of a population bottleneck on whooping crane mitochondrial DNA variation. Conservation Biology. 1999 Oct 23;13(5):1097-107. https://doi.org/10.1046/j.1523-1739.1999.97527.x

65. Groombridge JJ, Jones CG, Bruford MW, Nichols RA. 'Ghost'alleles of the Mauritius kestrel. Nature. 2000 Feb 10;403(6770):616-. https://doi.org/10.1038/35001148

66. Blanco G, Morinha F. Genetic signatures of population bottlenecks, relatedness, and inbreeding highlight recent and novel conservation concerns in the Egyptian vulture. PeerJ. 2021 Mar 25;9:e11139. https://doi.org/10.7717/peerj.11139

67. Keller LF, Jeffery KJ, Arcese P, Beaumont MA, Hochachka WM, Smith JN, et al. Immigration and the ephemerality of a natural population bottleneck: evidence from molecular markers. Proceedings of the Royal Society of London. Series B: Biological Sciences. 2001 Jul 7;268(1474):1387-94. https://doi.org/10.1098/rspb.2001.1607

68. McEachern MB, Van Vuren DH, Floyd CH, May B, Eadie JM. Bottlenecks and rescue effects in a fluctuating population of golden-mantled ground squirrels (Spermophilus lateralis). Conservation Genetics. 2011 Feb;12:285-96. https://doi.org/10.1007/s10595-010-0139-z

69. Jangjoo M, Matter SF, Roland J, Keyghobadi N. Connectivity rescues genetic diversity after a demographic bottleneck in a butterfly population network. Proceedings of the National Academy of Sciences. 2016 Sep 27;113(39):10914-9. https://doi.org/10.1073/pnas.1600865113

70. Heupink TH, van den Hoff J, Lambert DM. King penguin population on Macquarie Island recovers ancient DNA diversity after heavy exploitation in historic times. Biology letters. 2012 Aug 23;8(4):586-9. https://doi.org/10.1098/rsbl.2012.0053

71. Fleming CA, Baker AN. The Snares Western Chain. Notornis. 1973;20(1):37-45.

72. Sagar PM. Birds of the Western Chain Snares Islands, New Zealand. Ornithological Soc. of New Zealand; 1977.

73. Miskelly, CM, Sagar, PM, Tennyson, AJD, Scofield, RP. Birds of the Snares Islands, New Zealand. Notornis. 2001;48: 1–40.

74. Oehler DA, Marin M, Kusch A, Danielle L, Weakley LA, Fry WR. Foraging ranges in Southern Rockhopper Penguins (Eudyptes chrysocome chrysocome) on Isla Noir, Chile. International Journal of Avian and Wildlife Biology. 2018;3(4):320-5. https://doi.org/10.15406/ijawb.2018.03.00109

75. Thiebot JB, Bost CA. Exploitation of distant Antarctic habitats by juvenile macaroni penguins: conservation implications. https://doi.org/10.21203/rs.3.rs-3035279/v1

Supporting information

S1 Table. List of all samples and sampling locations included. Full list of Tawaki (*Eudyptes pachyrhynchus*), Erect-crested penguin (*Eudyptes sclateri*), and Eastern Rockhopper Penguin (*Eudyptes filholi*) samples included in the analysis.

Figure 1

Click here to access/download;Figure;fig1.tif

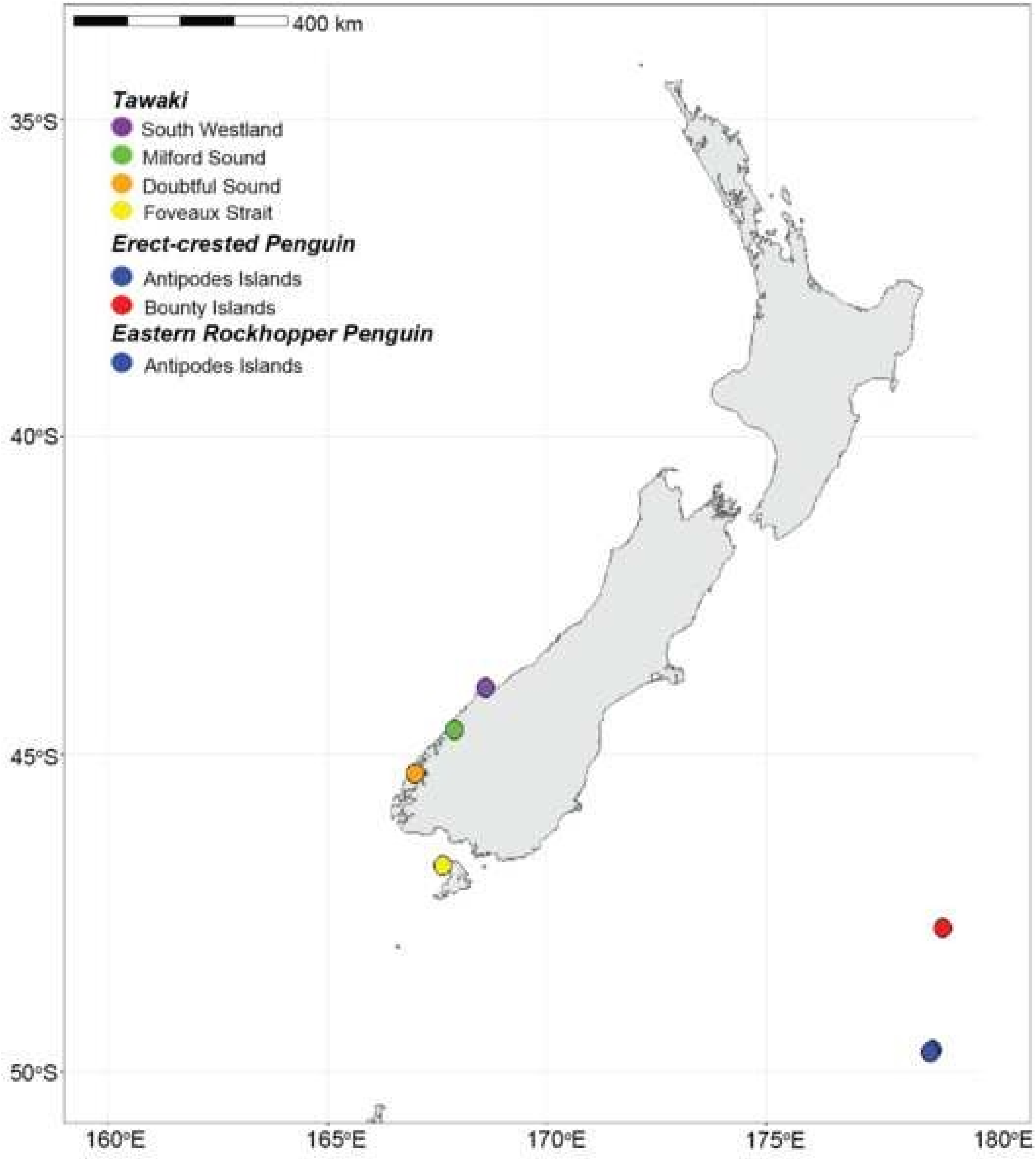

Figure 2

Click he

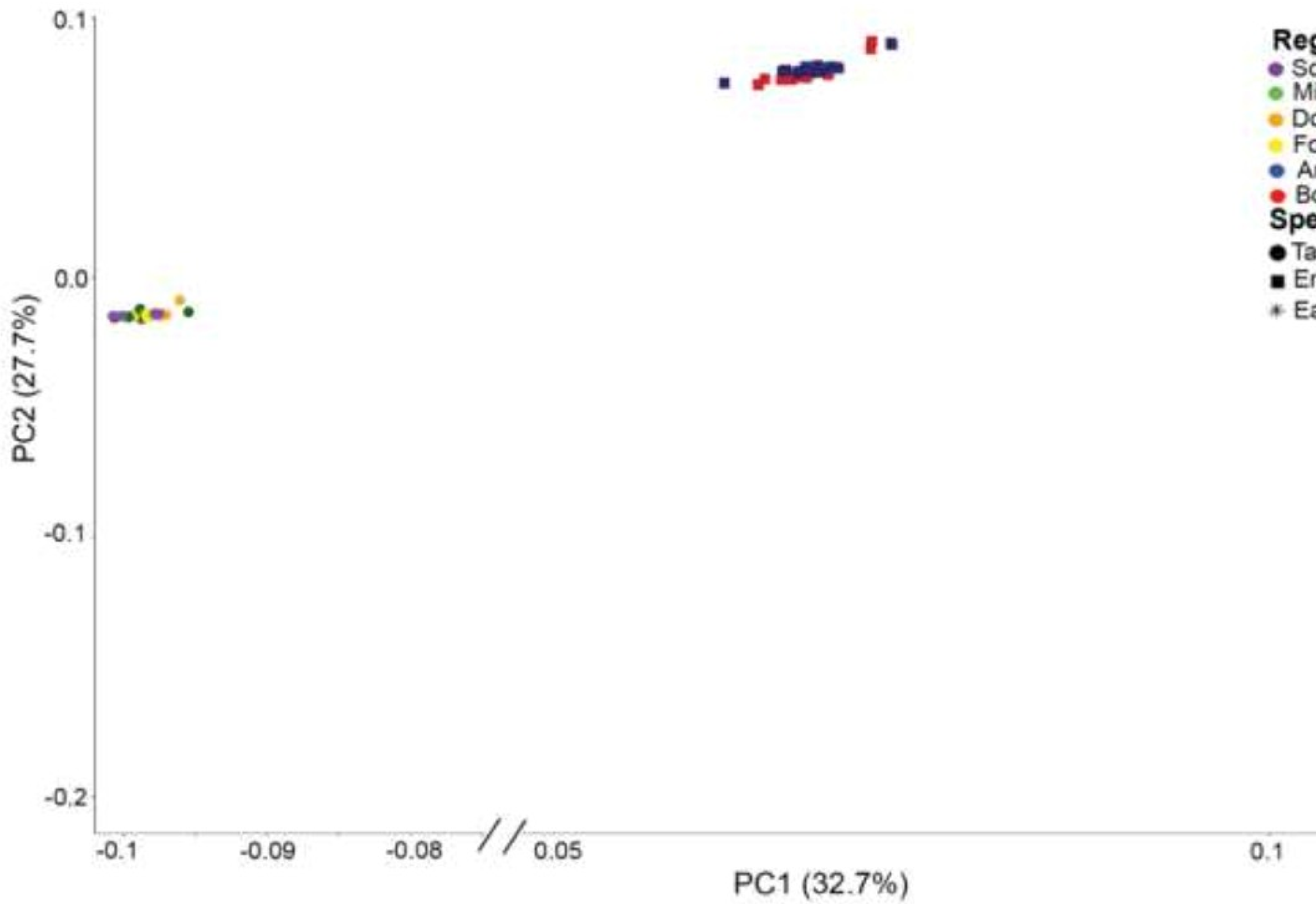

Figure 3

Click he

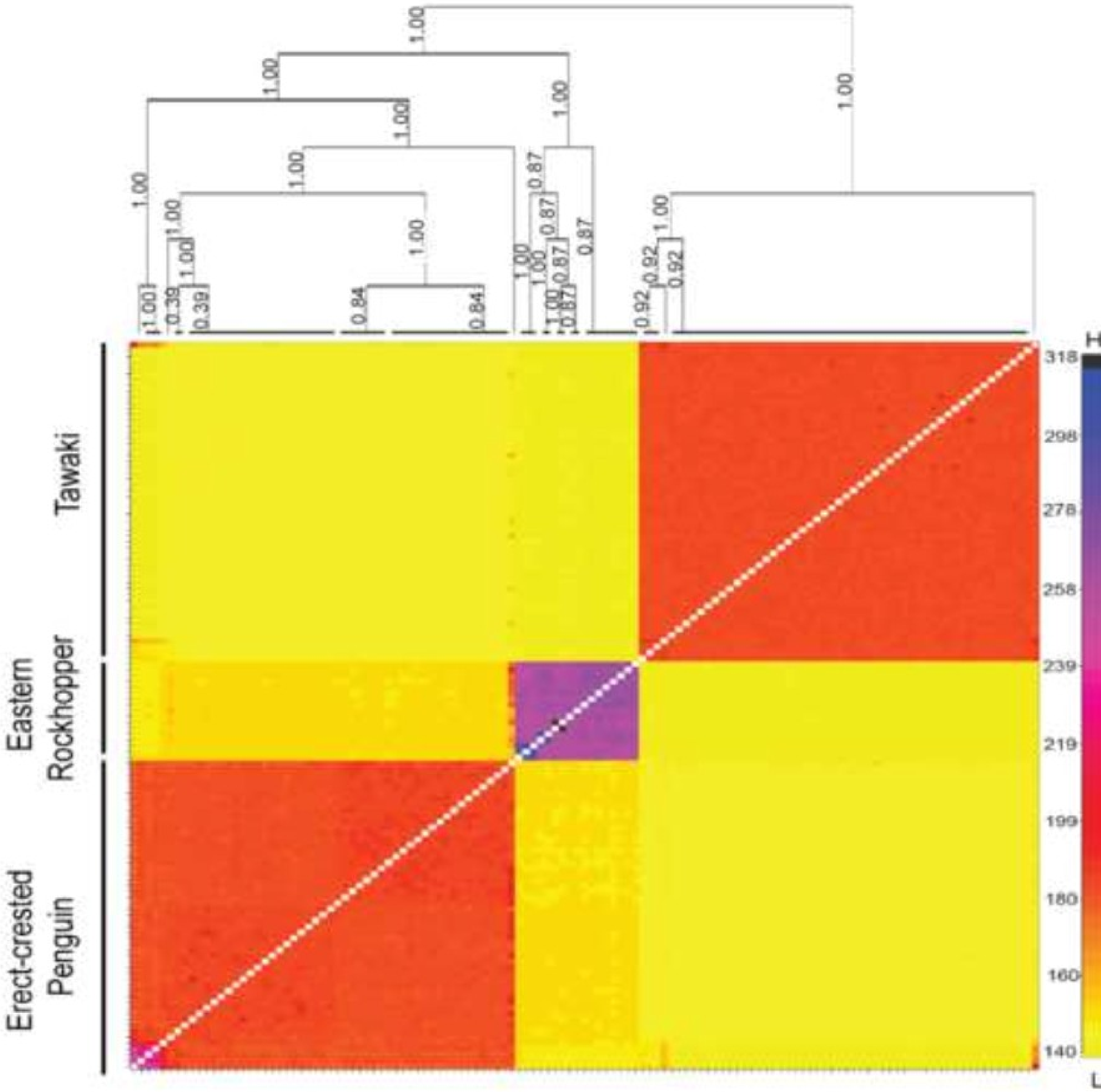

Figure 4

Click he

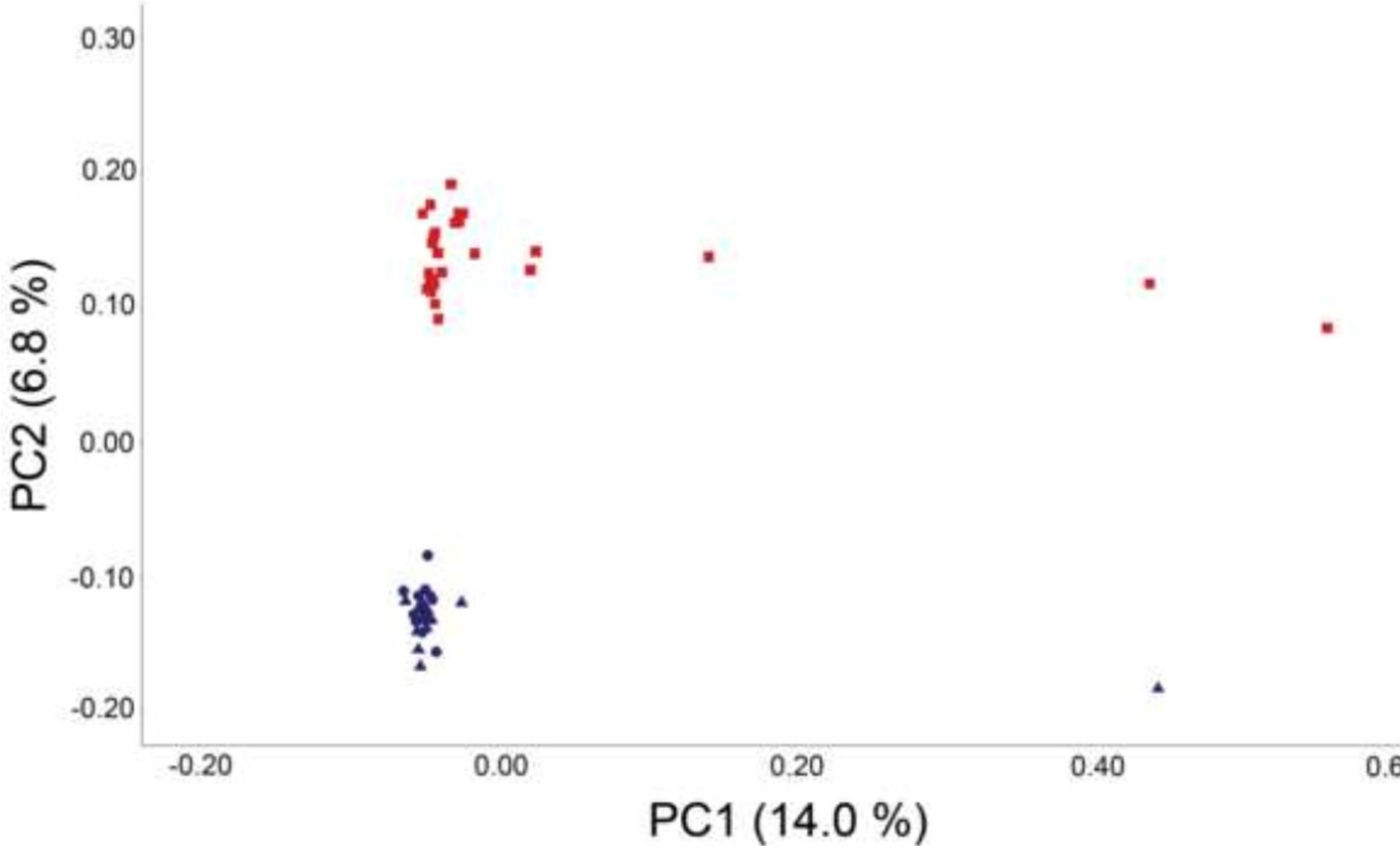

Figure 5

Click he

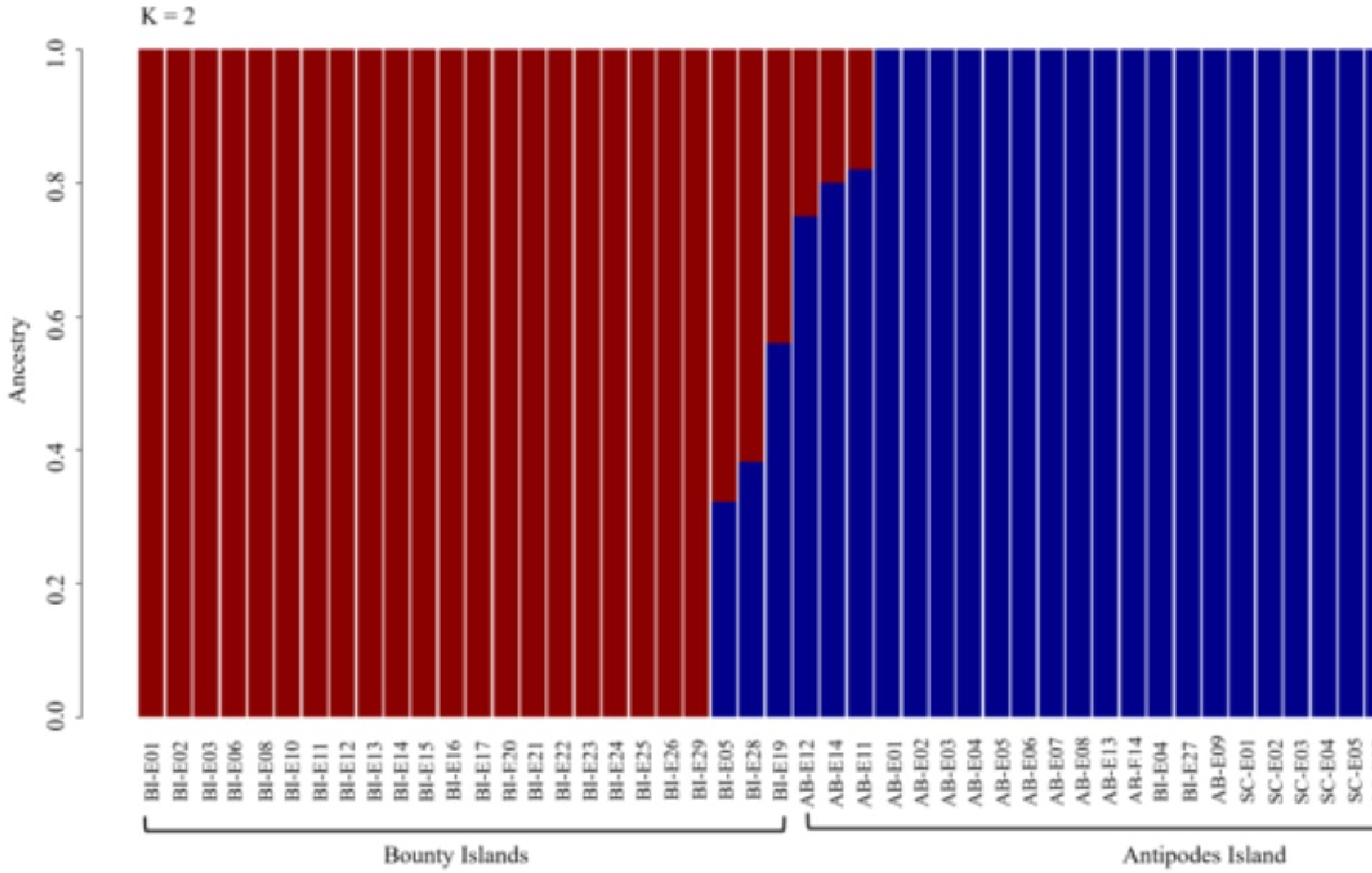

Supplemental Table

Click here to access/download
Supporting Information
white_supplemental_PLOSone_11_Sep_24.docx
