# Supplementary material for: Population structure of three New Zealand crested penguins identifies current conservation challenges for the Fiordland penguin/tawaki, erect-crested penguin, and eastern rockhopper penguin
Source: PLoS One. 2025 Aug 27;20(8):e0329545. doi: 10.1371/journal.pone.0329545 (PMC12385409; doi:10.1371/journal.pone.0329545)
Supplement: Attachment — Submitted filename: AL Crested penguins.docx [file pone.0329545.s003.docx]

The manuscript entitled ‘Population structure of three New Zealand crested penguins identifies current conservation challenges: Fiordland penguin/tawaki, erect-crested penguin, and Eastern rockhopper penguin’ investigated the population structure of three related crested penguin species in New Zealand using genome wide SNP data. Results showed panmixia among tawaki colonies, limited genetic diversity in eastern rockhopper penguins and significant genetic structure between two populations of erect-crested penguin exhibiting different patterns of foraging behaviour.

This study has a well-conceived biological question, especially regarding the long-distance movable seabirds. The data produced is astutely analyzed and the figures are well designed even though some of them could be improved (see further comments). Yet I have some concerns about the interpretation of the results. I have commented at greater length on this matter below and I think those ambiguities should be fixed and further clarified before the final acceptance of the paper.

**Introduction**

L62 – Please reword for clarity: (…) across all groups of seabirds and penguins are particularly vulnerable (…).

L64-L66 – The author introduced penguins as ‘particularly vulnerable species’ in L60-64 then started a new sentence with the importance of understanding the ecology and evolutionary of species. I would recommend replacing ‘species’ with ‘penguins’.

L64-L66 – Replace ‘vital’ with necessary/required or important.

L77-L79 – Please develop the case of king penguins. It is a wide-ranging seabird species, so the example may relate to IBD, but is there an overlap in foraging areas among populations? It is not clear how this example is related to the above descriptions of putative mechanisms of genetic divergence among seabird populations.

**Study system**

L81-L113 – The problematic is well explained and clear!

L119 – Please define ‘genetic histories’. Do you mean evolutionary histories?

L122 – Please replace ‘unstructured’ with ‘panmictic’.

L123 – Please replace ‘decreased’ with ‘low genetic diversity’.

L121-122 – Add at the end of the sentence that the genetic markers used in the present study are different from the mitochondrial DNA locus used in a previous study on the same species otherwise the reader does not understand how this study is different from the analyzes that have already been conducted (same in the section Conclusion).

**Material and methods**

L136 – For more clarity, : (…) of 2017, 2018, and 2022 divided into four regions: (…), and Foveaux Strait (Whenua Hou) (Fig1; S1 Table).’

**DNA extraction and ddRAD-Seq library preparation**

L155-L157 – It would be of interest to indicate which DNA samples were extracted with the DNeasy Blood and Tissue kit or with phenol-chloroform-isoamyl alcohol extractions.

**Results**

**Population structure/Summary statistics**

Admixture plots, PCA, FineRADstructure heatmaps and summary statistics ɸ_ST_ allowed coancestry assignments and detection of potential genetic clustering among populations and species. However, they do not take into consideration demographic histories of species – i.e. population trajectories or population trends. However, the author mentions several times those aspects throughout the manuscript (e.g. in the section Conclusion L366). I would find it interesting to build models that would give an idea of the evolution of the effective population size of populations (N_e_) through time as it may greatly affect the results – especially for erect-crested penguins. Indeed, while gene flow can be inferred from the amount of neutral allele frequency divergence among populations, deviation from the ‘island model’ of population structure following bottlenecks or population expansion may result in erroneous representation of contemporary gene flow based on population genetic clustering analyses.

In Table 2 & 3, it is not clear whether ɸ_ST_ and Tajima’D are significant or not.

L269 - L270 – The author argues that the high degree of coancestry might indicate a limited gene pool likely due to historical population declines. This implies that the populations might not be at mutation-drift equilibrium and hence that the results might be biased. This must be clarified.

In Fig 2 & 4 Symbols in plots could be bigger to help the reader.

**Discussion**

L289-L290 – The author concludes that population structure among tawaki populations is unlikely masked by recent population expansions. According to the previous considerations, the author should also conclude or at least mention that the tawaki case study does not support monogamy and philopatry as factors of genetic differentiation among seabird populations.

L318-L320 – I would discard this sentence as the author addresses it in L323-L326.

L331-L340 – I would be careful with the concluding remarks. The observation of highly structured erect-crested penguin populations might also reflect the size reduction of the antipode population. Current gene flow between colonies might occur but not be sufficient to overridden dramatic demographic history. The sample sizes for this species for Antipodes Islands are: Anchorage Bay N=14; South Coast N=14, which is quite low. I would recommend developing the demographic history aspect of erect-crested penguins in parallel to the allochrony explanation in the section discussion.

.
