# Supplementary material for: Population structure of three New Zealand crested penguins identifies current conservation challenges for the Fiordland penguin/tawaki, erect-crested penguin, and eastern rockhopper penguin
Source: PLoS One. 2025 Aug 27;20(8):e0329545. doi: 10.1371/journal.pone.0329545 (PMC12385409; doi:10.1371/journal.pone.0329545)
Supplement: Attachment — Submitted filename: white_comments_PLOSone_24_Apr_25.docx [file pone.0329545.s005.docx]

Reviewers' comments:

Reviewer's Responses to Questions

**Comments to the Author**

1. Is the manuscript technically sound, and do the data support the conclusions?

The manuscript must describe a technically sound piece of scientific research with data that supports the conclusions. Experiments must have been conducted rigorously, with appropriate controls, replication, and sample sizes. The conclusions must be drawn appropriately based on the data presented.

Reviewer #1: Yes

Reviewer #2: Yes

Reviewer #3: Yes

Reviewer #4: Partly

2. Has the statistical analysis been performed appropriately and rigorously?

Reviewer #1: Yes

Reviewer #2: Yes

Reviewer #3: Yes

Reviewer #4: Yes

3. Have the authors made all data underlying the findings in their manuscript fully available?

The [PLOS Data policy](http://track.editorialmanager.com/CL0/http:%2F%2Fwww.plosone.org%2Fstatic%2Fpolicies.action%23sharing/1/010f0192de170f80-afc651a4-099a-46a0-96e7-56f33611d7e4-000000/TEbGN4r1QsryXeUqzc4ZITQyzFPzqyEXWLAER5X-EWA=183) requires authors to make all data underlying the findings described in their manuscript fully available without restriction, with rare exception (please refer to the Data Availability Statement in the manuscript PDF file). The data should be provided as part of the manuscript or its supporting information, or deposited to a public repository. For example, in addition to summary statistics, the data points behind means, medians and variance measures should be available. If there are restrictions on publicly sharing data—e.g. participant privacy or use of data from a third party—those must be specified.

Reviewer #1: Yes

Reviewer #2: No

Reviewer #3: No

Reviewer #4: Yes

4. Is the manuscript presented in an intelligible fashion and written in standard English?

PLOS ONE does not copyedit accepted manuscripts, so the language in submitted articles must be clear, correct, and unambiguous. Any typographical or grammatical errors should be corrected at revision, so please note any specific errors here.

Reviewer #1: Yes

Reviewer #2: Yes

Reviewer #3: Yes

Reviewer #4: Yes

5. Review Comments to the Author

Please use the space provided to explain your answers to the questions above. You may also include additional comments for the author, including concerns about dual publication, research ethics, or publication ethics. (Please upload your review as an attachment if it exceeds 20,000 characters)

**Reviewer #1:** Overall the presented study adds to our current understanding of the genetic diversity and gene flow of the three species studied. The paper is nicely written, well laid out and analysed. I have some detailed comments on specific areas below:

Abstract – line 40 and 41 you introduce the species you are working on in this paper, but the next lines use tawaki to describe what was introduced as Fiordland, and adds sub-Antarctic to erect crested. I found it a little confusing and had to read the two again, so perhaps alter to improve consistency. **We modified the text to clarify and improve consistency. Tawaki is the Māori name is the recommended usage.**

Introduction
Under study system lines 81-82, you detail what the study is about, however, be clear that yours is looking at genetic population structure, and I do not think your study is looking at population trends in population size – please be clear that you are discussing genetic population structure and gene flow – as this paper cannot possible look at all these things together. You use historical published accounts to make sense of your genetic findings, but you do not present population trend data for example. **We updated the wording to be more clear about the purpose of the study. We have added analysis of Ne through time using a single-population model in dadi to address trends in population size. While gene flow has not been measured quantitatively, its presence/absence is revealed by the other results such as Fst and admixture.**

Figure 1 map – why are the two species on the Antipodes islands in the same colour? I find the key confusing – the colours are for locations, but then you have listed species. **We chose to color code by location rather than by species to be consistent with the color coding of the PCA and admixture plots. The distinction between colony location (island) is crucial to the interpretation of the results and the discussion.**

Fig 2. I dislike the colours and the shapes here – they are not matching the key and are confusing. The red and blue are much darker on the plot than the key and the Eastern Rockhopper star shape is also a different blue to the island colour in the key. Spelling error of Eastern Rockhopper in the key (Roockhopper). **We updated the key to ensure that all of the color shades match exactly with the figure itself. The star shape for the rockhoppers is the same shade of blue as is used for the erect-crested penguins and in the key. We would like to keep this color palette. We corrected the typo in Rockhopper.**

Conclusion: Something is a little lacking in the conclusion – perhaps further details on how or what researchers could investigate to understand why the Antipodes population is in such decline, or what additional genetic research could add to our understanding or aiding species recovery. Perhaps relate back to the introduction and initial aims and reasoning as to why you carried out the study in the first instance. I just feel it could be stronger to emphasise the important results you have found. **We added additional discussion of conservation efforts for each of these species into the Conclusion. The Ne through time plots now added to the manuscript also confirm a population decline for erected-crested penguins.**

**Reviewer #2:** This is a very important research regading genetic diversity of three penguin species using a robust genetic technique and a well-performed bionformatics pipeline.
There are some important missing details in the introduction and methodology, and some of the paragraphs of the discussion must be improved in order to clarify the authors' ideas. All my considerations were inserted as notes in the marked pdf.

I agree with both of the ending sentences of the abstract, but I don’t see them connected at all; firstly, because the authors mentioned that tawaki populations are probably increasing, and then they argue that conservation efforts should prioritize the protection of marine habitats. But why, if this species’ populations are increasing?

I suggest the authors to rewrite this conclusion so the conservation aspects are linked to the decreasing populations/populations with low genetic diversity.

Furthermore, the authors have never mentioned the bycatch issue before, and then the abstract conclusion also mentions the importance of reducing fisheries mortality. I suggest the major threats are called before so they can be mentioned in the possible solutions. **While the tawaki population is currently doing well, conservation efforts such as protecting marine habitats are still crucial for safeguarding this species. This includes protecting marine habitats in the breeding season as well as during non-breeding periods. Without protection of foraging areas and management of fisheries in these regions, tawaki may become imperiled like so many other penguin species. We have updated the text to clarify.**

L92-93: I think it would be valuable to give a background in the introduction about these "modern DNA sequencing methods" and their advantages **We have added a brief rationale for choosing these techniques and cited a good review paper that goes into detail of the strengths of various methods for avian conservation.**

L114: This term is commonly used to refer to genetic predisposition to some diseases. If refering to eventual bottlenecks and other phenomena, I suggest you to use some other term such as "population genetics features" **We updated in the text for clarity.**

L120: I think it would be better to use (bio)geography. **We updated this in the text.**

L152: It's essential, at least for this species, to discern how many samples were collected at Antipodes and how many at Bounty. But please, add this information for all species and colonies **We added sample sizes per site to the text for clarity.**

Fig 3: Tick marks in both horizontal and vertical axis represent individual samples **We updated in the text for clarity.**

L315: So, it was a bottleneck, right? I wasn't able to understand the construction of this paragraph: firstly, the authors mention that population bottlenecks are cited to explain high levels of coancestry; then, I understand that they argued that it couldn't be a bottleneck because if it was, population size would have already rebounded. In the end, they suggest that it was a sharp decline - so, was it a bottleneck or not? Why? I suggest that this whole paragraph would be rewritten to clarify the authors' ideas.

**There is no expectation that diversity should recover after a bottleneck, except in the case where immigrant populations still harbor high diversity (i.e., regional vs. global bottleneck). To help clarify what has happened in these species we have added Ne through time plots. Only tawaki has increased, eastern rockhoppers and erect-crested penguins have both declined accordingly to these analyses, which also indicate the timeframe over which population size changes have occurred.**

L347-352: I don't understand why the authors cite this example because it seems like it will end with a similar explanation (i.e. the Snares penguin would also exhibit an asynchronous annual cycle probably caused by an oceanic front). As the cause of this phenomenon is probably not the same, I think the authors could cite another species whose phenotypic/genotypic features are influenced by oceanic fronts - as they do in the next paragraph - such as Calonectris borealis/diomedea - https://doi.org/10.3354/meps07974

**While we appreciate this comment, we believe that the inclusion of the Snares Penguin example is pertinent to understanding the processes occurring with the Erect-crested Penguin. The main Snares group and the Western Chain are separated by only about 5km of ocean and there no frontal zones separating these two groups. Yet, they show a similar breeding asynchrony as the Erect-crested penguins (which are separated by about 200km and differences in access to the subtropical front). We decided to include the Snares Penguin example as an alternative scenario where the frontal zone is not a barrier to gene flow or otherwise influencing the behavior of the population. In the Erect-crested penguins, we also have a scenario that is not the typical frontal zone or oceanic basin separating two populations as is often seen in other seabirds. Instead, we have two island groups both located on the Campbell Plateau, in the subantarctic zone, south of the subtropical front but with distinct behaviors and genetics. As there is no marine division between them, it is likely that the location of the Bounty Island closer to the subtropical front allows them to travel northward to forage rather than southward as on the Antipodes. Therefore, we have a scenario with the frontal zone influencing behavior rather than separating basins and populations. This shows the need for additional considerations in interpreting differences in phenology as does the case of the Snares Penguin.**

**Reviewer #3:** The manuscript entitled ‘Population structure of three New Zealand crested penguins identifies current conservation challenges: Fiordland penguin/tawaki, erect-crested penguin, and Eastern rockhopper penguin’ investigated the population structure of three related crested penguin species in New Zealand using genome wide SNP data. Results showed panmixia among tawaki colonies, limited genetic diversity in eastern rockhopper penguins and significant genetic structure between two populations of erect-crested penguin exhibiting different patterns of foraging behaviour.

This study has a well-conceived biological question, especially regarding the long-distance movable seabirds. The data produced is astutely analyzed and the figures are well designed even though some of them could be improved (see further comments). Yet I have some concerns about the interpretation of the results. I have commented at greater length on this matter below and I think those ambiguities should be fixed and further clarified before the final acceptance of the paper.

**Introduction**

L62 – Please reword for clarity: (…) across all groups of seabirds and penguins are particularly vulnerable (…). **We reworded the text for clarity.**

L64-L66 – The author introduced penguins as ‘particularly vulnerable species’ in L60-64 then started a new sentence with the importance of understanding the ecology and evolutionary of species. I would recommend replacing ‘species’ with ‘penguins’. **We reworded the text for clarity.**

L64-L66 – Replace ‘vital’ with necessary/required or important. **We reworded the text for clarity.**

L77-L79 – Please develop the case of king penguins. It is a wide-ranging seabird species, so the example may relate to IBD, but is there an overlap in foraging areas among populations? It is not clear how this example is related to the above descriptions of putative mechanisms of genetic divergence among seabird populations. **We believe that including an example of the effect of dispersal (long distance between discrete colonies) on genetic diversity is important for the context of our findings. We suggest in the results that dispersal ability in tawaki is an important factor in the lack of structure across their range (similar to the king penguins) and a lack of dispersal is likely a contributing factor in the decline of Eastern rockhopper populations and low genetic diversity on Antipodes Island. We have reworded the text to increase the clarity and relevance of the reference.**

**Study system**

L81-L113 – The problematic is well explained and clear!

L119 – Please define ‘genetic histories’. Do you mean evolutionary histories? **We updated the text for clarity.**

L122 – Please replace ‘unstructured’ with ‘panmictic’. **We updated the text with this suggestion.**

L123 – Please replace ‘decreased’ with ‘low genetic diversity’. **We updated the text with this suggestion.**

L121-122 – Add at the end of the sentence that the genetic markers used in the present study are different from the mitochondrial DNA locus used in a previous study on the same species otherwise the reader does not understand how this study is different from the analyzes that have already been conducted (same in the section Conclusion). **We updated the text for clarity.**

**Material and methods**

L136 – For more clarity, : (…) of 2017, 2018, and 2022 divided into four regions: (…), and Foveaux Strait (Whenua Hou) (Fig1; S1 Table).’ **We updated the text for more clarity.**

**DNA extraction and ddRAD-Seq library preparation**

L155-L157 – It would be of interest to indicate which DNA samples were extracted with the DNeasy Blood and Tissue kit or with phenol-chloroform-isoamyl alcohol extractions. **We added clarifying information to the text and a new column to Table S1 to indicate which method was used for each sample.**

**Results**

**Population structure/Summary statistics**

Admixture plots, PCA, FineRADstructure heatmaps and summary statistics ɸ_ST_ allowed coancestry assignments and detection of potential genetic clustering among populations and species. However, they do not take into consideration demographic histories of species – i.e. population trajectories or population trends. However, the author mentions several times those aspects throughout the manuscript (e.g. in the section Conclusion L366). I would find it interesting to build models that would give an idea of the evolution of the effective population size of populations (N_e_) through time as it may greatly affect the results – especially for erect-crested penguins. Indeed, while gene flow can be inferred from the amount of neutral allele frequency divergence among populations, deviation from the ‘island model’ of population structure following bottlenecks or population expansion may result in erroneous representation of contemporary gene flow based on population genetic clustering analyses.

**Unfortunately, the population assessments for both the erect-crested and Eastern rockhopper penguins is highly uncertain. We are currently working on the first population assessment for the erect-crested penguin across its range. The data from prior assessments of the islands individually have been thrown into question recently as the methods used are often vague at best (see Mattern et al., Erect-crested penguins on the Bounty Islands: population size and trends determined from ground counts and drone surveys. Notornis. 2021 Jan1;68(1):37-50 and Mattern, T., & Wilson, K. J. (2018). New Zealand penguins–current knowledge and research priorities. *A report compiled for Birds New Zealand*.).**

**As stated above, we have added Ne through time plots. Only tawaki has increased, eastern rockhoppers and erect-crested penguins have both declined accordingly to these analyses, which also indicate the timeframe over which population size changes have occurred.**

L269 - L270 – The author argues that the high degree of coancestry might indicate a limited gene pool likely due to historical population declines. This implies that the populations might not be at mutation-drift equilibrium and hence that the results might be biased. This must be clarified.

**Yes, this would be expected, but drift-mutation equilibrium are not required for the these analyses that were undertaken. e.g., drift-mutation equilibrium would be required to extract Ne from Fst but not to calculate Fst. Similarly, the Ne through time plots are obtained from the site-frequency spectrum, not a coalescent model.**

In Fig 2 & 4 Symbols in plots could be bigger to help the reader. **We increased the size of the symbols on the plots but unfortunately they merge into an unreadable blob. So, we decided to use the current size.**

**Discussion**

L289-L290 – The author concludes that population structure among tawaki populations is unlikely masked by recent population expansions. According to the previous considerations, the author should also conclude or at least mention that the tawaki case study does not support monogamy and philopatry as factors of genetic differentiation among seabird populations. **We updated the text to include this consideration.**

L318-L320 – I would discard this sentence as the author addresses it in L323-L326. **The first sentence is in reference to Eastern rockhoppers while the second is referring to erect-crested penguins.**

L331-L340 – I would be careful with the concluding remarks. The observation of highly structured erect-crested penguin populations might also reflect the size reduction of the antipode population. Current gene flow between colonies might occur but not be sufficient to overridden dramatic demographic history. The sample sizes for this species for Antipodes Islands are: Anchorage Bay N=14; South Coast N=14, which is quite low. I would recommend developing the demographic history aspect of erect-crested penguins in parallel to the allochrony explanation in the section discussion. **Similar to the previous comment, we just don’t have reliable data on the demographics of rockhoppers or erect-crested penguins on Antipodes Island. The estimates we used in the paper are the best published estimates but they are also lacking in many ways. Therefore, we do not feel that we currently have the data available to accurately develop a case for the demographic history of these species.**

Reviewer #4: This manuscript investigates genetic connectivity, structure, and diversity in three penguin species in New Zealand: Fiordland penguin (tawaki), erect-crested penguin, and Eastern rockhopper penguin. Using ddRADseq SNP data, the authors examined population structure across different colonies, focusing on conservation implications given observed population declines.

The article is very well-written, with clear background on each of the species and a clear set of testable hypotheses to be explored presented in the introduction and finishes with a nicely rounded discussion. The genetic results support known demographics of the species and populations. I have a few comments which I think will improve the statistical validity and interpretation of the results. I recommend additional analyses, for which the authors can decide how necessary they are. However, I think with such a nice dataset, pushing it just a little further in regards to such analyses, will help support the author’s key messages.

Main comments:

Line 202: fineRADstructure uses the full haplotype information (i.e. every SNP on the RAD locus). Please confirm whether all SNPs were used or if the LD pruned dataset (described previously) was used. This can quite dramatically change the results of fineRADstructure so please be sure of this.

**The LD pruned dataset was used.**

Line 268-269 (also lines 308-309). I would recommend checking via other methods (e.g. vcftools –relatedness2 option) to see if these two samples really do represent siblings. Looking at your fineRADstructure plot, it looks to me as though there may be other related individuals in this sample too. Related to this, you should remove one member of the any related pair before calculating the pop. gen summary statistics.

**This is a focus of our next paper, including more recent sampling.**

Line 232: I can’t see obvious “hybrids” in your PCA. Please elaborate. **Here we meant ‘hybrids’ between the two genetic clusters meaning individuals with genes from both the Bounty and Antipodes clusters. We have removed the term in the text for clarity.**

In Fig. 4 (PCA of erect-crested penguins), it’s curious to me that PC1 (14%) separates individuals sampled from both colonies, whereas it is PC2 (6.8 %) that differentiates the colonies. What’s going on along PC1? Are the samples lower-coverage? Lower genetic diversity? Please investigate these outlier samples. If there is not data quality reason to remove these individuals, then please discuss this result. **The outlier individuals corresponded to those that had both Bounty and Antipodes ancestry. Coverage was similar over all samples.**

Line 270: yes to population declines, but also isolation and genetic drift (see my comments below regarding gene flow and effective population size analyses).

In the fineRADstructure plot, is there a reason for the Eastern rockhopper sharing higher ancestry with the Erect-crested penguin? This would be worth discussing

**This is expected to happen if the rockhoppers and erect-cresteds have high co-ancestry compared to the tawaki.**

Given the large weight placed on gene flow between colonies placed in the discussion, a formal analysis of gene flow would be a nice addition. i.e. using BayesAss (<https://github.com/brannala/BA3>). See <https://onlinelibrary.wiley.com/doi/full/10.1111/ddi.13399> for a nice use of this in rockhopper penguins. Such an analysis is relatively straightforward and would add an analytical framework to much of what is currently discussed.

**We feel that an analysis of gene flow is an excellent suggestion, but better suited for a separate paper, for example among the tawaki colonies where gene flow does occur. The samples of erected crested penguins and rockhoppers are too limited in distribution in our opinion.**

I would also love to see estimates of the effective population size, especially for tawaki and erect-crested penguins as the sample sizes are more than high enough for performing these estimates. Providing these estimates would substantially help the author’s claims about the different changes in demography and in interpreting the population structure and genetic diversity estimates. Using for example: <https://onlinelibrary.wiley.com/doi/10.1111/1755-0998.13890>
**We have added an analysis of Ne through time using a dadi model for each species and for the two island groups of erect-crested penguin.**

Minor comments:

In the keywords, I would suggest using ddRADseq (capitalised RAD) as in the rest of the manuscript. **We updated this error.**

Line 45: instead of “thousands of autosomal loci” can you be more quantitative?

**We have updated the wording. The actual # is presented in the results.**

Line 171: cite the genome paper? <https://doi.org/10.1093/gigascience/giz117>
**We did not use this paper as a reference for developing the methods described.**

Line 171-172. You probably used the mem algorithm of bwa. Specify this
**Yes, this is the default algorithm for aligning reads to the reference genome.**

Line 200: hyphenate “species-specific” **We updated in the text.**

Line 263: “lower differentiation” **We updated in the text.**

Line 270: remove the word “Calculating” **We omitted this in the text.**

I would recommend using a fewer number of decimal places for some of the statistics reported in Table 2 to increase readability. **We have updated the reporting by rounding to two decimal places.**

The Figures are very low resolution, meaning I couldn’t interpret, for example, the PCA presented in Figure 2. Please replace with a higher-resolution figure. **We have included high resolution figures.**

6. PLOS authors have the option to publish the peer review history of their article ([what does this mean?](https://track.editorialmanager.com/CL0/https:%2F%2Fjournals.plos.org%2Fplosone%2Fs%2Feditorial-and-peer-review-process%23loc-peer-review-history/1/010f0192de170f80-afc651a4-099a-46a0-96e7-56f33611d7e4-000000/gDFnNB5XEDn8Pc6FVLMygAE1cOm_tEyN7626Wr0sbG4=183)). If published, this will include your full peer review and any attached files.


If you choose “no”, your identity will remain anonymous but your review may still be made public.


**Do you want your identity to be public for this peer review?** For information about this choice, including consent withdrawal, please see our [Privacy Policy](https://track.editorialmanager.com/CL0/https:%2F%2Fwww.plos.org%2Fprivacy-policy/1/010f0192de170f80-afc651a4-099a-46a0-96e7-56f33611d7e4-000000/olwJNLy6wyu7IZt0wp8SoelHPPmDz2g1o2S2XFrCYLE=183).

Reviewer #1: No

Reviewer #2: No

Reviewer #3: No

Reviewer #4: No
