# Supplementary material for: Population structure of three New Zealand crested penguins identifies current conservation challenges for the Fiordland penguin/tawaki, erect-crested penguin, and eastern rockhopper penguin
Source: PLoS One. 2025 Aug 27;20(8):e0329545. doi: 10.1371/journal.pone.0329545 (PMC12385409; doi:10.1371/journal.pone.0329545)
Supplement: Attachment — Submitted filename: white_comments_16_June_2025.docx [file pone.0329545.s006.docx]

**Response to Reviewer Comments**

**PONE-D-24-41700R1**

*Population structure of three New Zealand crested penguins identifies current conservation challenges for the Fiordland penguin/tawaki, erect-crested penguin, and Eastern rockhopper penguin*

**We would like to thank the reviewers for their time and many helpful comments throughout this review process. Please find our comments to the suggested revisions below.**

Review Comments to the Author

Please use the space provided to explain your answers to the questions above. You may also include additional comments for the author, including concerns about dual publication, research ethics, or publication ethics. (Please upload your review as an attachment if it exceeds 20,000 characters)

Reviewer #2: The authors mostly addressed the suggestions, corrections and questions of the reviewers. I believe this is a well-conducted study on genetic diversity and connectivity that highlights the importance of conservation measures directed toward evolutionarily significant units; for these reasons, it is worthy of publication in PLOS One.

Some final considerations:
In L49-50, I think that Eastern rockhopper penguin should be mentioned beside the erected-crested penguins. In the next sentence, when you speak directly of the Eastern rockhopper population of Antipodes Islands, it seems disconnected because you haven’t mentioned the other species previously.
**We thank the reviewer for this comment, however we do not agree that the eastern rockhopper should also be mentioned in this sentence. This statement is referencing our finding of population structure in erect-crested penguins (only) based on breeding island group. We did not include eastern rockhoppers in this same statement as they do not occur on both island groups and therefore mentioning them in this sentence could be misleading. This structure is the primary finding we wish to convey about erect-crested penguins while low genetic diversity is the primary finding for eastern rockhoppers. We also do mention eastern rockhoppers earlier in the abstract in the same (and only) sentence that introduced the erect-crested penguins.**

L81-82: I agree with Reviewer #3 when he stated that “it is is not clear how this example is related to the above descriptions of putative mechanisms of genetic divergence among seabird populations.” Despite the rewording of this sentence, I still feel like it is disconnected to the aforementioned possibilities. The authors stated that “We suggest in the results that dispersal ability in tawaki is an important factor in the lack of structure across their range (similar to the king penguins) and a lack of dispersal is likely a contributing factor in the decline of Eastern rockhopper populations and low genetic diversity on Antipodes Island.” I understand the connection of the example of the King Penguins to the authors’ results, but it doesn’t aggregate much to mention a different species in which this happens before the reader even knows what your results are going to be (in the introduction). I think that maybe the authors should just mention this possibility as just another one, without giving it a highlight with the King Penguins example.

**We have removed the mention of king penguins here and replaced the citation with a more general metanalysis instead to provide a broader example of the mechanism at this stage of the paper.**

In L132-134, the authors state that “Finally, we investigated whether the presence of the STF, and associated higher primary productivity, influences gene flow between the Antipodes Islands and Bounty Islands populations of erect-crested penguins.” I am concerned that the authors did not, in fact, investigate the influence of different levels of primary productivity on the genetic structuring between the two populations. Rather, they discuss the possibility of this phenomenon, providing examples of other species in which this has been observed. In order to investigate the influence of primary productivity as a selective pressure, authors should have obtained chlorophyll-α levels in the foraging areas of each population, and then tested this association (or influence) through Mantel tests or Latent Factor Mixed Models (LFMMs), for example. Some examples of works that did, in fact, test this association are Nunes & Bugoni (2017) - <https://onlinelibrary.wiley.com/doi/abs/10.1111/jbi.13142> ; Mazzochi et al. (2024) – <https://link.springer.com/article/10.1007/s10592-024-01613-x> .
If the authors are willing to do this, I believe it would be an interest analysis that could provide a more robust answer to their research question. However, if they are not, the referenced sentence should be reworded – avoiding the phrasing that frames this as an analysis, and instead using terms such as “discussed”, “considered the possibility”, or similar.

**We thank the reviewer for this suggestion. While we do agree that adding environmental data (chlorophyll-a and SST especially) would be very interesting, we believe it is out of the scope and desired purpose for this paper. However, we are working on another paper that does look more in depth into the divergent ecology of the erect-crested penguin populations. Therefore, we have updated the wording of this sentence to avoid unnecessary confusion.**

L352-353: I still don’t understand why the authors mention that population size and genetic diversity would rebound if there were modest levels of immigration. Are they arguing that there is, in fact, no immigration? But what evidences show that there is/there is not immigration? As they give the example of the king penguins that recovered pre-bottleneck levels and then don’t make a link with the study case, I don’t understand why they mention the importance of immigration for this situation.
**In this case we are making the point that the eastern rockhopper population on Antipodes is likely very isolated. If there was immigration from other populations of eastern rockhoppers (i.e., from Campbell, Auckland, or Macquarie) then we would expect genetic diversity to be higher. But what we do need is sampling from other populations to quantify gene flow among them and assess the status of this species at other sites. This is exactly the focus of our expanded work onto these islands starting in the 2025 field season. We have reworded the line to clarify.**

In L362-364, the authors also say that a little gene flow could be existing from other colonies to the Antipodes Islands, which reduces chances of genetic recovery. But shouldn’t it increase the chances, as the authors previously stated? Or have they made an attempt to argue that this “little gene flow” wouldn’t be enough to rebound pre-bottleneck levels of genetic diversity?

**We appreciate the reviewer pointing out this confusing wording. By “little gene flow” we meant that there is potentially a lack of gene flow, not that there is a small amount of gene flow. We have updated for clarity.**

Finally, it appears that the authors have not provided a link to the available genomic data in the manuscript (data availability statement?). I believe this is an important detail that should be included.

**We have uploaded the data to the NCBI SRA repository to be available upon publication at BioProject ID PRJNA1277195.**

Reviewer #3: The authors have adequately addressed my comments raised in a previous round of review and I feel that this manuscript is now acceptable for publication

**We thank the reviewer for their comments and efforts to help us improve the quality of this paper.**
